# Effects of Dimerization on the Deacylase Activities of Human SIRT2

**DOI:** 10.1021/acs.biochem.3c00381

**Published:** 2023-11-15

**Authors:** Jie Yang, Nathan I. Nicely, Brian P. Weiser

**Affiliations:** † Department of Molecular Biology, Rowan University School of Osteopathic Medicine, Stratford, New Jersey 08084, United States; ‡ Department of Pharmacology, University of North Carolina at Chapel Hill, Chapel Hill, North Carolina 27599, United States

## Abstract

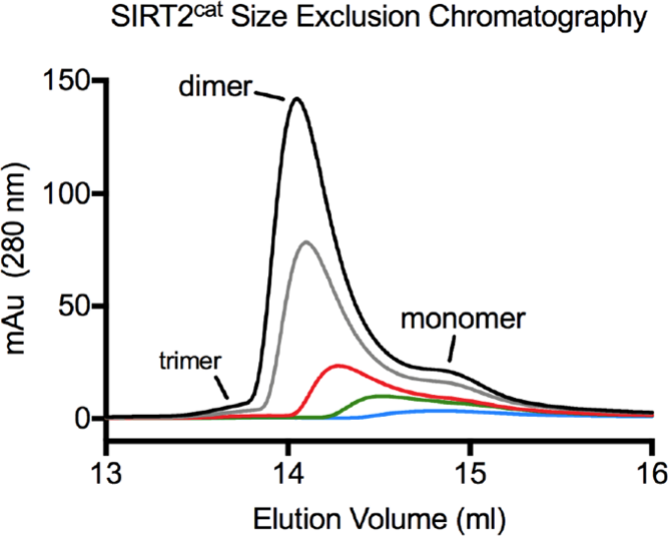

Human sirtuin isoform 2 (SIRT2) is an NAD^+^-dependent enzyme that functions as a lysine deacetylase and defatty-acylase. Here, we report that SIRT2 readily dimerizes in solution and in cells and that dimerization affects its ability to remove different acyl modifications from substrates. Dimerization of recombinant SIRT2 was revealed with analytical size exclusion chromatography and chemical cross-linking. Dimerized SIRT2 dissociates into monomers upon binding long fatty acylated substrates (decanoyl-, dodecanoyl-, and myristoyl-lysine). However, we did not observe dissociation of dimeric SIRT2 in the presence of acetyl-lysine. Analysis of X-ray crystal structures led us to discover a SIRT2 double mutant (Q142A/E340A) that is impaired in its ability to dimerize, which was confirmed with chemical cross-linking and in cells with a split-GFP approach. In enzyme assays, the SIRT2(Q142A/E340A) mutant had normal defatty-acylase activity and impaired deacetylase activity compared with the wild-type protein. These results indicate that dimerization is essential for optimal SIRT2 function as a deacetylase. Moreover, we show that SIRT2 dimers can be dissociated by a deacetylase and defatty-acylase inhibitor, ascorbyl palmitate. Our finding that its oligomeric state can affect the acyl substrate selectivity of SIRT2 is a novel mode of activity regulation by the enzyme that can be altered genetically or pharmacologically.

## Introduction

Lysine acyl modifications are prevalent on proteins that control many aspects of cellular physiology. Human sirtuin isoform 2 (SIRT2) is an NAD^+^-dependent enzyme that functions as a lysine deacylase. By catalyzing the removal of protein acyl modifications, SIRT2 regulates diverse processes including cancer growth, neurodegeneration, metabolism, and inflammation.^1−6^ The acyl substrates of SIRT2 are chemically diverse and range from small modifications such as acetyl-lysine to much larger, fatty modifications such as myristoyl-lysine.^7^ Numerous studies have characterized the substrate-specific enzymology of SIRT2.^7−13^ Small molecules are being developed to selectively modulate SIRT2’s deacylase activities to understand how different acylations affect disease states and to explore SIRT2’s potential as a therapeutic target.^9,14−17^ Additionally, novel acyl modifications on lysines continue to be identified as SIRT2 substrates,^8,18−21^ which increases the challenge of modulating select SIRT2 activities and increases our need for tools that can probe specific SIRT2 functions.

Despite the importance of SIRT2’s enzymatic activity in cells, an uncertain aspect of its character is whether SIRT2 oligomerizes, and how oligomerization could affect its function. A seminal study that identified SIRT2 as an epigenetic eraser purified the enzyme from *Escherichia coli* and HEK293 cells, and estimated that SIRT2 had a size that was consistent with a homotrimer.^22^ However, SIRT2 has since been treated as a monomeric protein in characterizing its reaction mechanism and substrate selectivity.^7−13^ Interestingly, a variety of SIRT2 homo-oligomers have been observed in the crystal structures of the enzyme (Table S1). The crystallographic oligomers are heterogeneous and show different protein orientations and different contacts between adjacent SIRT2 molecules, which may or may not be visible in the asymmetric unit. In crystals, there is not a clear relationship between SIRT2’s oligomeric state and the presence or absence of specific acyl modifications or ligands that were cocrystallized with the protein with the exception of an autoinhibited SIRT2 dimer that forms in the presence of ADP-ribose (Table S1). It is unclear whether these oligomeric states are biologically relevant or an artifact of the crystallization process, which relies on excessively high protein concentrations and nonphysiological salts and precipitants.

In this work, we report our observations that SIRT2 does indeed oligomerize in solution, favoring a homodimer at concentrations greater than ∼100 nM. We also demonstrate that SIRT2 can dimerize in human cells. The SIRT2 dimer typically escapes detection during chromatographic purification of the enzyme and was revealed to us during analytical size exclusion chromatography (SEC) and chemical cross-linking experiments. Interestingly, we found that dimerized SIRT2 dissociates into monomers upon binding long fatty acyl substrates such as myristoyl-lysine, but SIRT2 remains dimerized when bound to smaller substrates such as acetyl-lysine. Our analysis of crystallographic SIRT2 oligomers led us to produce a SIRT2 mutant that is defective in dimerizing and has reduced deacetylase activity compared to that of the wild-type protein, but this SIRT2 mutant retains its normal demyristoylase activity. This indicates that the oligomeric state of SIRT2 could influence its different deacylase activities. Finally, we provide evidence that small molecules targeting SIRT2 can be used to alter its oligomeric state in solution and in cells.

## Materials and Methods

### Synthetic Peptides

All peptides were obtained from New England Peptide. 13mer synthetic peptides containing the histone H4 sequence with Lys16 modified were used in cross-linking reactions and were described previously (H4K16 peptide sequence: KGLGKGGAK(Acylation)RHRK).^9^ 16mer synthetic peptides containing an identical sequence with additional residues on the C-terminus were used in MALDI-based enzyme activity assays and were also described previously (sequence: KGLGKGGAK(Acylation)RHRKGWW).^9,14^ The FAM-myristoyl-H4K16 peptide used in cross-linking, binding, and crystallography experiments contained the 13mer sequence; a fluorescein (FAM) group was attached to the N-terminal amine of the peptide with a PEG4 linker in between, and the peptide had a C-terminal carboxylic acid. The 28mer SIRT2 peptide corresponding to amino acids 286–313 contained the sequence NKEKAGQSDPFLGMIMGLGGGMDFDSKK with no modifications to the peptide.

### Production of Recombinant SIRT2 Proteins

The production of recombinant SIRT2 catalytic domain (SIRT2^cat^) from Addgene plasmid #102622 was described previously.^4,9^ The plasmid encoded an N-terminal 6xHis-tag and SUMO domain fused in-frame to SIRT2^cat^, and these N-terminal tags were removed by the SUMO protease ULP1 during the protein purification procedure.^9,23^ The final SIRT2^cat^ protein contained amino acids 34–356 with a single extra serine on the N-terminus (residue numbering from UniProt #Q8IXJ6-1). SIRT2^cat^ has an actual molecular weight (MW) of 36 576 Da, and its solution concentrations were determined from its absorbance at 280 nm and its calculated extinction coefficient (32 890 M^–1^cm^–1^).^24^ Bacterial expression and purification of full-length SIRT2 (amino acids 1–389; UniProt #Q8IXJ6-1) was performed identically to SIRT2^cat^ and provided similar yield.^9^ To construct the plasmid for full-length SIRT2 expression, the N- and C-terminal portions of the gene were separately amplified from a mammalian full-length SIRT2 expression vector (Addgene plasmid #102623)^4^ and inserted into the 6xHis-SUMO-SIRT2^cat^ plasmid using standard QuikChange/mega-primer methods. Full-length SIRT2 has an actual MW of 43 182 Da.

SIRT2^cat^(Q142A/E340A) was produced by mutating the two residues with standard QuikChange methods in the 6xHis-SUMO-SIRT2^cat^ plasmid. The mutant was expressed in a manner identical to that of the wild-type protein. The mutant protein was purified over a Ni^2+^ column, and the 6xHis-SUMO tag was removed. The mutant protein was then purified with MonoQ chromatography prior to SEC. All cloning was verified with Sanger sequencing.

### SEC Analysis of SIRT2 and Estimation of Affinity for Self-Association

SEC was performed using a Bio-Rad NGC chromatography system with a Bio-Rad Enrich SEC 650 column (10 mm × 300 mm). The SEC standards were from Sigma-Aldrich (catalog #69385), and PBS with 1 mM DTT was used as the mobile phase and as the buffer to dilute the standards and SIRT2^cat^.

For analytical SEC analyses that observed SIRT2^cat^ dimer and monomer peaks, the injection volume was 100 μL, and the injected SIRT2^cat^ concentration ranged from 2 to 80 μM. Although we injected SIRT2^cat^ concentrations between 2 and 80 μM through the SEC column, proteins become substantially diluted during SEC and ultimately elute at much lower concentrations. The final concentrations of SIRT2 dimer and monomer that eluted off the column could be determined from the absorbance values at the height of the elution peaks (λ = 280 nm) and SIRT2’s extinction coefficient.^9^ For example, the large dimer peak that resulted from analyzing 80 μM SIRT2^cat^ had a concentration of only 4.3 μM when eluting off the column based on its peak UV–vis absorbance at 280 nm and the protein’s extinction coefficient,^9^ whereas the monomer peak had a concentration of 0.6 μM. We reasoned that these elution concentrations could be used to determine a *K*_d_ for SIRT2’s interaction with itself if we assume that the monomer and dimer were at equilibrium on the SEC column prior to passing through a UV–vis detector. The *K*_d_ of SIRT2 for self-association was described by the equation
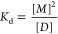
1where monomer and dimer are represented as molar concentrations.^28^ Every SEC run that resulted in two identifiable peaks (monomer and dimer) could be used to calculate a *K*_d_, and the *K*_d_ that we estimated in the Discussion section was the average from all of the SEC runs.

For qualitative SEC analysis of 52 μM SIRT2 in the presence and absence of FAM-myristoyl-H4K16 peptide, the injection volume was 142.5 μL. For SEC analysis of purified full-length SIRT2, 56 μM protein was injected in a 100 μL volume.

### SIRT2 Cross-Linking Reactions

Cross-linking reactions (without SIRT2 inhibitors) were performed at room temperature for 1 h in PBS containing 1 mM DTT. The reaction volume was 12 μL, and the protein/peptide/ADP-ribose mixtures were equilibrated for ∼5 min before the addition of cross-linker. The primary cross-linking reagent we used was Bis(NHS)-PEG5 from Thermo Scientific (catalog #21581), which was used at a final concentration of 1 mM. The cross-linking reactions were quenched by adding 2 μL of a solution that contained 250 mM Tris, 1.92 M glycine, and 1% SDS followed by boiling for 1 min. In some experiments, formaldehyde (proteomics grade from VWR) was used as the cross-linker at a concentration of 0.125–1%. These reactions were also quenched with 2 μL of 250 mM Tris, 1.92 M glycine, and 1% SDS, but the samples were only heated to 50 °C for 5 min to avoid reversing the cross-link. 2 μL of 80% glycerol was then added to all cross-linked samples before the reactions were separated by sodium dodecyl-sulfate polyacrylamide gel electrophoresis (SDS-PAGE). After SDS-PAGE, an Azure imager was used for fluorescent imaging when applicable, and all gels were stained with Coomassie blue. Following Coomassie blue staining, the intensities of SIRT2 monomer, dimer, and trimer bands were quantified using Fiji/ImageJ.^25^ There was small variability in the level of wild-type SIRT2 protein that cross-linked as an oligomer in different experiments; for this reason, when discussing changes in the amount of cross-linked oligomer caused by mutations or ligands, we only make comparisons to the wild-type protein that was cross-linked and analyzed on the same gel as the mutated or treated SIRT2.

Cross-linking reactions in the presence of SIRT2 inhibitors were performed with Bis(NHS)-PEG5 essentially as described above with minor modifications. In these experiments, the reaction volume was 50 μL, and each reaction had a final DMSO concentration of 2% because of its use as the solvent for dissolving the inhibitors. DMSO alone was added to the cross-linked control (no inhibitor) samples that were analyzed on the same gels as the inhibitor-containing samples and had no effect on the level of oligomeric SIRT2. The final concentrations of SIRT2 inhibitor added during initial cross-linking experiments were chosen based on their IC_50_ values for inhibiting SIRT2 deacetylase activity *in vitro* to ensure that a large fraction of SIRT2 was bound to ligand during cross-linking. The IC_50_ values for inhibiting SIRT2 deacetylase activity are as follows: thiomyristoyl (TM), 0.03–0.04 μM;^4,15^ SirReal2, 0.14–0.16 μM;^15,26^ ascorbyl palmitate, 3–17 μM;^14^ pictilisib, ∼3 μM;^14^ propofol, 140 μM.^9^ For this work, TM and SirReal2 were obtained from Selleck Chemicals, and our use of ascorbyl palmitate, pictilisib, and propofol was reported previously.^9,14^

### SIRT2^cat^ Structural Analyses and Comparisons

Protein structures were visualized and graphically represented using PyMOL.^27^ The methods used to analyze and predict the oligomeric state of SIRT2^cat^ in existing X-ray crystal structures, as reported in Table S1, are presented in the Supporting Information. Molecular contacts that occurred between adjacent apo-SIRT2^cat^ molecules that were found in the asymmetric unit of PDB code 3ZGO were manually identified in PyMOL with the aid of the Measurement tool; atoms from different apo-SIRT2^cat^ molecules that were within 4 Å of each other were considered to be in contact. The structure and position of residues found at the interfaces of apo-SIRT2^cat^ molecules in the asymmetric unit of 3ZGO were compared to the same residues in our crystal structure of SIRT2^cat^ bound to myristoylated peptide in the following manner. 3ZGO (which contained three SIRT2^cat^ molecules) and three copies of our crystal structure (each containing one SIRT2^cat^ molecule) were loaded into the same PyMOL window. Each copy of our structure was aligned to one of the apo-SIRT2^cat^ molecules using only the Rossmann fold domain residues for the alignment (amino acids 79–83, 161–166, 256–260, 282–286, and 317–321). Relative to the Rossmann fold domain, it was visually apparent that myristoyl peptide binding did not change the position of residues at Interface 1 (defined in the Results section), but peptide binding did change the position of residues that comprised Interface 2. The tilt of the C-terminal helix of SIRT2^cat^ on its hinge was measured using the “AngleBetweenHelices” script in PyMOL by drawing the best-fit vectors through the α carbons of helix residues 326–334 and helix residues 338–355.

### Enzyme Activity Assays

SIRT2^cat^ deacylase assays were performed using a previously described MALDI-MS method.^14^ All reactions were performed at 37 °C in PBS with 1 mM DTT. With the exception of the deacetylase reactions that determined the *K*_m, NAD+_, all other assays were performed with 1 mM NAD^+^. Enzyme concentrations for the reactions were as follows: deacetylase assays, 40 nM; de-4-oxononanoylase assays, 100 nM; dedecanoylase assays, 50 nM; dedodecanoylase assays, 50 nM; demyristoylase assays, 50 nM.

Based on the enzyme concentrations that we used in each deacylase assay, the estimated percent of SIRT2^cat^ that existed as a dimer in the reactions were as follows: deacetylase assays, 18.5%; de-4-oxononanoylase assays, 30.5%; dedecanoylase assays, 21.2%; dedodecanoylase assays, 21.2%; demyristoylase assays, 21.2%. This was calculated by considering the equation
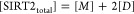
2where [SIRT2_total_] is the total molar amount of enzyme used in a given reaction, [*M*] is the concentration of monomer present, and [*D*] is the concentration of dimer present. The equation can be rearranged such that
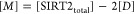
3and eq 3 can be combined with eq 1 such that
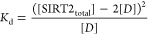
4[SIRT2_total_] is a known value for each reaction and *K*_d_ is the dissociation constant for the self-association of SIRT2^cat^, which we estimated to be 121 nM. Thus, solving for [*D*] gives the concentration of SIRT2^cat^ dimer in each enzyme reaction. Similar mathematical rearrangements can be used to solve for [*M*] in each reaction. The percent of SIRT2^cat^ that existed as a dimer in each reaction was then determined using the equation
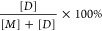
5and a similar equation with [*M*] in the numerator could be used to determine the percent of SIRT2^cat^ that existed as a monomer in each reaction.

### Cellular Split-GFP Experiments

The split GFP system that reported the dimerization of full-length SIRT2 in cells was constructed in the following manner. The gene for full-length SIRT2 was obtained from an Addgene plasmid (#102623).^4^ The gene was amplified with restriction enzyme sites on the N-terminus (BsiWI) and C-terminus (NotI) that were used with restriction enzyme cloning to insert the SIRT2 gene into an empty mammalian expression vector (pIRESneo3, Takara catalog #631621). The genes encoding the N-terminal and C-terminal GFP fragments were also obtained from Addgene (plasmids #40729 and #40730).^28^ The N-terminal GFP fragment was amplified from the Addgene plasmid and inserted onto the N-terminus of SIRT2 in the mammalian expression vector using the QuikChange/mega-primer method; an eight-residue linker separated the N-terminal GFP fragment and SIRT2. Separately, the C-terminal GFP fragment was amplified and inserted onto the C-terminus of SIRT2 on a separate plasmid using the QuikChange/mega-primer method; a six-residue linker separated SIRT2 and the C-terminal GFP fragment. Thus, we obtained two mammalian expression plasmids: (1) N-terminal GFP fragment fused to the N-terminus of full-length SIRT2 and (2) C-terminal GFP fragment fused to the C-terminus of full-length SIRT2. Point mutations (Q142A and E340A) were made on the SIRT2-fusion plasmids using QuikChange to study the effects of mutations on SIRT2 dimerization in cells. For the negative control condition where we expressed the N-terminal GFP fragment and C-terminal GFP fragment simultaneously without them being fused to SIRT2, the genes were amplified and inserted into empty pIRESneo3 vectors using standard restriction enzyme cloning as before with BsiWI and NotI sites. All plasmid sequences were verified with Sanger sequencing, and the full sequences of the GFP fragment-fused SIRT2 proteins can be found in the Supporting Information.

A549 cells were maintained in DMEM with 10% FBS supplemented with penicillin-streptomycin. 25 000 cells were seeded and left for 16 h in Corning transfectagro media (catalog #40–300-CV) on top of a poly-d-lysine-coated coverslip that was placed in a 6-well plate. The two split GFP-SIRT2 plasmids were combined in an equal molar ratio. The plasmids (0.25 μg each) were cotransfected into the A549 cells using Promega ViaFect reagent (catalog #E4981). After 24 h transfection, the transfectagro media was removed and replaced with fresh DMEM containing 10% FBS, and the cells grew for another 24 h (this 48 h post-transfection time point was shown in the main text figure). The cells were then washed twice with PBS followed by a 10 min fixation using 4% paraformaldehyde in PBS. The cells were washed twice with PBS, treated with 0.2% Triton X-100 for 15 min, then washed briefly with PBS and finally water. The excess water on the coverslip was briefly dried by tapping the coverslip edge on a paper towel, and then the coverslip was mounted on a glass slide using mounting medium that contained DAPI (Vector Laboratories, catalog #H-2000). Slides were imaged using a Keyence BZ-X710 fluorescence microscope with the following filter settings: DAPI, excitation/emission was 300–400/438–484 nm; GFP, excitation/emission was 448/500–550 nm. After obtaining the cell images under identical conditions, fluorescence intensities of at least six randomly selected cells on each coverslip were quantified with Fiji/ImageJ without any manipulation to the images.^25^ Relative fluorescence intensity was calculated as the ratio of the GFP/DAPI intensities.

Experiments examining the effects of ascorbyl palmitate on SIRT2 dimerization in cells were performed essentially as described above with minor modifications. The two split GFP-SIRT2 plasmids were cotransfected into A549 cells in transfectagro media as before. After 24 h transfection, the media was removed and replaced with fresh DMEM containing 10% FBS and 200 μM ascorbyl palmitate. The cells grew for another 6 h before the media was removed, the cells were washed with PBS, then fixed with paraformaldehyde and processed as described above. We note that a 6 h treatment with 200 μM ascorbyl palmitate was previously shown to inhibit SIRT2 deacetylase and defatty-acylase activities in other cancer cell lines with minimal toxicity at that time point.^14^ Because ascorbyl palmitate was originally dissolved in DMSO, the cells were also exposed to 0.2% DMSO during the 6 h treatment; DMSO alone at this concentration was also added to control cells during the experiment.

### Estimation of SIRT2 Concentration in Cells

Wiśniewski et al. determined the protein copy number of SIRT2 in four different cell lines (HepG2, A549, PC-3, and U87MG cells).^29^ The protein copy number values from the four cell lines were converted to moles of SIRT2 per cell assuming a mass of 43 182 Da per SIRT2 molecule. The moles of SIRT2 per cell were divided by the approximate cytoplasmic volume of a cell (0.94 pL, estimated from HeLa cells).^30,31^ The estimated concentration determined for SIRT2 in each cell line was between 30 and 100 nM.

## Results

### Oligomeric States of SIRT2 in Solution

**Figure 1:**
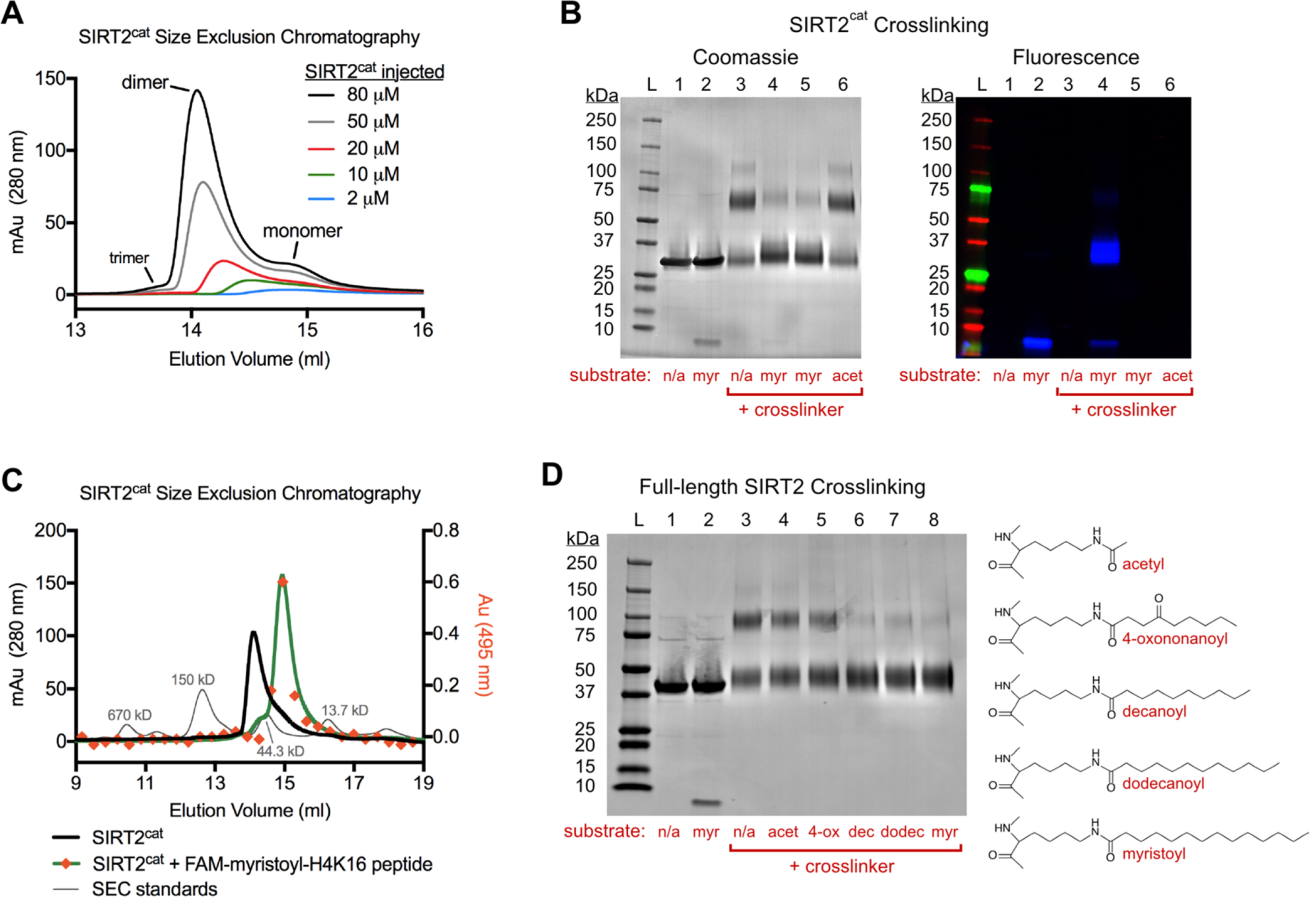
SIRT2 dimerizes and transitions to monomer upon myristoyl substrate binding. (A) Analytical SEC chromatograms were collected after injecting 100 μL of the indicated SIRT2^cat^ concentrations. UV–vis absorbance (in milli-Absorbance units (mAu)) was measured by a detector in line with the SEC column. (B) SIRT2^cat^ cross-linking experiment with Bis(NHS)-PEG5 as visualized by SDS-PAGE. Cross-linker was omitted from lanes 1 and 2, and all lanes contained 10 μM SIRT2^cat^. Additional components for each lane include: (1) SIRT2^cat^ alone; (2) 12 μM FAM-myristoyl-H4K16 peptide; (3) SIRT2^cat^ alone; (4) 12 μM FAM-myristoyl-H4K16 peptide; (5) 12 μM myristoyl-H4K16 peptide; (6) 12 μM acetyl-H4K16 peptide. The fluorescence image was taken before staining the gel with Coomassie blue and reimaging. (C) Analytical SEC chromatograms for SIRT2^cat^, the SIRT2^cat^—FAM-myristoyl-H4K16 peptide complex, and SEC standards. In addition to having the in-line UV–vis detector, 0.34 mL fractions were collected during the run with the SIRT2^cat^/peptide complex, and the absorbance of each fraction at 495 nm was separately measured with a spectrophotometer to observe elution of the FAM-labeled peptide (right *y* axis and orange diamonds). (D) Full-length SIRT2 cross-linking experiment with Bis(NHS)-PEG5 as visualized by SDS-PAGE. Cross-linker was omitted from lanes 1 and 2, and all lanes contained 10 μM SIRT2. 12 μM acylated H4K16 peptide was included in lanes 4–8 as indicated.

Using standard expression and purification procedures, the SIRT2 catalytic domain (SIRT2^cat^) (amino acids 34–356) can be purified from bacteria with good yield (>1 mg of purified enzyme per L of *E. coli* culture).^4,11,12,32^ In our lab, SEC is used as the final step for the purification of SIRT2^cat^.^9^ When several milligrams of SIRT2^cat^ are purified with SEC, we routinely observe a single dominant protein peak that we confirm with SDS-PAGE to be the desired protein (Figure S1).^9^ In contrast, here we used analytical SEC to analyze lower amounts of recombinant SIRT2^cat^ (<300 μg). For the initial experiments, we analyzed multiple concentrations of SIRT2^cat^ with SEC and compared its elution to SEC standards under identical chromatography conditions. The SIRT2 chromatograms were dominated by two peaks, which do not resolve when large amounts of protein are analyzed (Figure 1A). The largest peak we observed after injecting 80 μM SIRT2^cat^ through the column had an elution volume of 14.1 mL and an apparent MW of 56 kDa, while the smaller peak had an elution volume of 14.9 mL and an apparent MW of 32 kDa. The actual MW of SIRT2^cat^ was 37 kDa, which led us to suspect that the two peaks were dimeric and monomeric forms of the enzyme. Both peaks were present when we injected lower concentrations of SIRT2^cat^ except when 2 μM protein was analyzed, where SIRT2 appeared to be monomeric (Figure 1A).

Next, we used covalent cross-linking to confirm that the dominant state of SIRT2^cat^ at higher protein concentrations was indeed dimer. Here, we used a symmetric cross-linker that contained an NHS ester on both sides of a flexible PEG5 linker (Bis(NHS)-PEG5). In the absence of cross-linker, 10 μM SIRT2^cat^ migrated on SDS-PAGE with an apparent MW of 31 kDa (Figure 1B, lane 1). When SIRT2^cat^ alone was treated with cross-linker, the majority of the protein (50.8% of the total) was 62 kDa on SDS-PAGE, which was consistent with a dimer (Figure 1B, lane 3). Lower levels of cross-linked SIRT2^cat^ also migrated at MWs that corresponded to monomer (43.2%) or trimer (6.0%) (Figure 1B, lane 3) (see Table S2 for quantification of protein bands from all cross-linking gels). After visualizing the cross-linking gels, we suspected that less than 5% of SIRT2^cat^ may have existed as a trimer at the highest protein concentration that we analyzed with SEC (Figure 1A). We also repeated SIRT2^cat^ cross-linking experiments using formaldehyde instead of the NHS ester-based reagent. Formaldehyde cross-linking also showed that SIRT2 dimerizes in solution, indicating that this result was independent of cross-linker chemistry (Figure S2).

To test whether SIRT2^cat^ would remain dimerized while performing its deacylase reactions, we cross-linked SIRT2^cat^ in the presence of myristoylated or acetylated substrate peptides that had a sequence derived from Histone H4 with lysine 16 modified (“H4K16”).^22,33,34^ Interestingly, SIRT2^cat^ was almost entirely monomeric in the presence of the myristoyl-H4K16 peptide (Figure 1B, lane 5), but SIRT2^cat^ remained mostly an oligomer in the presence of the acetyl-H4K16 peptide (Figure 1B, lane 6). Although SIRT2^cat^ has a higher affinity for myristoyl substrates compared to acetyl substrates,^8,11^ we determined that at least 25% of SIRT2^cat^ was bound to acetyl-H4K16 peptide in that cross-linking experiment based on a published binding assay (Figure S3),^14^ yet the dimeric population of SIRT2^cat^ was unchanged. We also ruled out the possibility that SIRT2^cat^ could transition to monomer in the presence of both acetyl peptide and ADP-ribose, which might promote a SIRT2 conformational change by serving as a nonhydrolyzable NAD^+^ mimic (Figure S4).^9,35^ ADP-ribose alone also had no effect on SIRT2^cat^, which still favored a dimer in its presence (Figure S4).

The experiments above strongly indicated that the interaction of SIRT2^cat^ with the myristoyl modification on the H4K16 peptide was essential for driving the dimer to monomer transition. To better visualize this, we cross-linked SIRT2^cat^ while bound to a fluorescein-labeled, myristoyl-H4K16 peptide called “FAM-myristoyl-H4K16”. This fluorescent myristoyl peptide also caused a SIRT2^cat^ transition from dimer to monomer (Figure 1B, lane 4). We also mixed SIRT2^cat^ and FAM-myristoyl-H4K16 peptide at 52 μM each and analyzed their complex with SEC, which confirmed that the protein/peptide complex was monomeric (Figure 1C). FAM-myristoyl-H4K16 peptide and the unlabeled myristoyl-H4K16 peptide had nearly identical affinity for SIRT2^cat^ using a gel shift assay (*K*_d_ = 1–2 μM) (Figure S5), and SIRT2^cat^ processes FAM-myristoyl-H4K16 peptide as a conventional myristoyl substrate (Figure S5). Moreover, we cocrystallized SIRT2^cat^ with FAM-myristoyl-H4K16 peptide and solved its X-ray structure, which revealed a SIRT2^cat^-myristoyl peptide complex similar to published structures of SIRT2^cat^ bound to other myristoyl peptides (root-mean-square deviation of 0.7 Å when aligned to PDB code 4Y6L or 4R8M) (Figure S5 and Table S3).^10,12^ Thus, this fluorescent myristoyl peptide and its cocrystal structure bound to SIRT2^cat^ are useful for exploring SIRT2^cat^ interactions with myristoylated substrate.

SIRT2^cat^ lacks 33 residues on its N-terminus and an additional 33 amino acids on its C-terminus. The short terminal segments of SIRT2 are thought to be disordered and are often omitted during enzymology studies because they could reduce expression yield and antagonize SIRT2 crystallization,^36^ but recombinant full-length SIRT2 was reported to be stable.^32^ We expressed full-length SIRT2 in *E. coli* and purified the enzyme to learn more about its oligomeric states (Figure 1D, lane 1). When cross-linked, ∼50% of full-length SIRT2 was oligomeric, which was similar to its catalytic domain (Figure 1D, lane 3, and Table S2). Dimerized full-length SIRT2 also underwent a clear transition to monomer when bound to the myristoyl substrate (Figure 1D, lane 8). We examined how interactions with other acyl modifications affect the enzyme’s oligomeric state. We determined that binding long acyl chains such as decanoyl and dodecanoyl also promoted a dimer to monomer transition for full-length SIRT2. However, the nine-carbon 4-oxononanoyl modification was much less effective at dissociating dimers and only increased the monomeric population of SIRT2^cat^ by 11% (Figure 1D). Finally, on analytical SEC, full-length SIRT2 primarily eluted as a dimer with an apparent MW of 68 kDa, and a smaller amount of monomer was observed at 43 kDa (Figure S6); the actual MW of full-length SIRT2 is 43 kDa.

### Identification of Residues That Disrupt SIRT2 Dimerization

**Figure 2:**
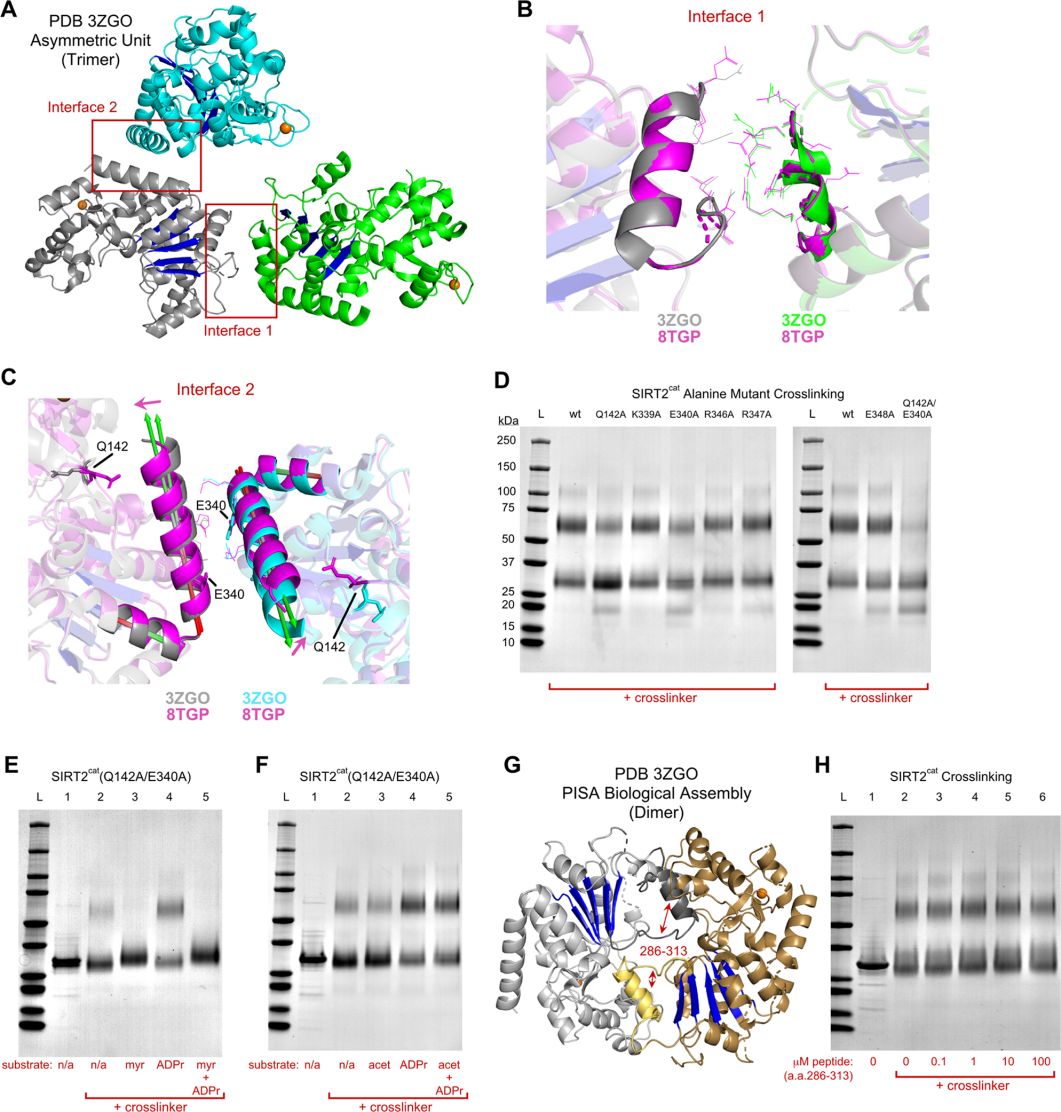
Identification of residues that disrupt SIRT2 dimer formation. (A) Orientation of three SIRT2^cat^ molecules that were present in the asymmetric unit of the apo-SIRT2^cat^ crystal structure (PDB code 3ZGO). The gray SIRT2^cat^ molecule contacts the green and cyan SIRT2^cat^ molecules at different interfaces. The Rossmann fold domain of each molecule is colored blue, and the zinc ion is colored orange. (B) Interface 1 between gray and green SIRT2^cat^ molecules as shown in (A). Additionally, our structure of SIRT2^cat^ bound to myristoyl peptide (PDB code 8TGP), shown in magenta, was aligned using the Rossmann fold domain to each SIRT2^cat^ molecule. Protein secondary structure and side chain positions at Interface 1 were similar between those of apo-SIRT2^cat^ and myristoyl substrate-bound SIRT2^cat^. (C) Interface 2 between gray and cyan SIRT2^cat^ molecules as shown in (A), along with our structure of SIRT2^cat^ bound to myristoyl peptide shown in magenta, which were aligned through the Rossmann fold domains. Upon myristoyl substrate binding, the long C-terminal helix of residues 338–355 tilted 4° on its hinge toward the catalytic domain to contact residue Q142, which also changed its position. The direction of the tilt was indicated by magenta arrows. (D) Cross-linking experiment with Bis(NHS)-PEG5 and 10 μM SIRT2^cat^ which contained alanine mutations at residues along the Interface 2 helix. Q142A and E340A mutations weakened dimer formation. Note that the faint band at ∼20 kDa was a minor contaminant that was chromatographically removed for experiments in (E) and (F). (E) Cross-linking experiment with 10 μM SIRT2^cat^(Q142A/E340A). 12 μM myristoyl peptide stabilized the monomer, but 300 μM ADP-ribose alone promoted dimer formation. (F) Cross-linking experiment showing that, in contrast to ADP-ribose, acetyl peptide did not influence the oligomeric state of SIRT2^cat^(Q142A/E340A). (G) Dimeric SIRT2^cat^ biological assembly predicted by PISA using the apo-SIRT2^cat^ crystal structure as input (PDB code 3ZGO). Key interface residues 286–313 were dark gray and yellow, and these belong to SIRT2^cat^ molecules colored light gray and brown, respectively. The small helices at the interface contain residues 295–304 which are flanked by short disordered segments (Figure S7). (H) Cross-linking experiment showing that a synthetic peptide of SIRT2 residues 286–313 did not affect the oligomeric state of SIRT2.

Crystallographic dimers of SIRT2^cat^ were heterogeneous and did not suggest a specific protein–protein interface responsible for homo-oligomerization (Table S1). We observed SIRT2 dimerization in solution in the absence of substrate or ligand (Figure 1), so we focused our analysis on the crystal structure of apo-SIRT2^cat^ (PDB code 3ZGO).^36,37^ Apo-SIRT2^cat^ formed an asymmetric trimer with two distinct protein–protein interfaces in the asymmetric unit (Figure 2A).^36,37^ We defined Interface 1 as between the bases of two Rossmann fold domains from different protein molecules, and we defined Interface 2 as forming between the C-terminal helices of different protein molecules (Figure 2A). Neither interface contained extensive protein–protein interactions; Interface 1 contained a salt bridge, two additional hydrogen bonds, and two C–O contacts, whereas Interface 2 contained two hydrogen bonds and one C–O contact (Table S4). Neither interface was strongly suggested as being responsible for dimerization based on this analysis.

Instead, we hypothesized that the position of interface residues should change between the structures of apo-SIRT2^cat^ and myristoyl peptide-bound SIRT2^cat^ if that interface was altered during substrate binding and was important for the dimer to monomer transition. Thus, we aligned our crystal structure of SIRT2^cat^ bound to myristoyl peptide to each molecule of the apo-SIRT2^cat^ trimer to observe structural differences at Interface 1 or Interface 2. We used the β-strand residues from the Rossmann fold domain for the alignment because these residues are rigid and hardly change between apo-SIRT2^cat^ and substrate-bound structures (root-mean-square deviation < 0.15 Å) (see the Materials and Methods section). The backbone atoms near Interface 1 overlapped perfectly in the aligned structures and did not change their position when SIRT2^cat^ was substrate-bound (Figure 2B). On the other hand, the C-terminal helix at Interface 2 tilted inward 4° on its hinge when the protein was bound to myristoyl peptide (Figure 2C), suggesting Interface 2 had a structural change that could affect protein dimerization.

We generated six recombinant SIRT2^cat^ proteins with single-point mutations at residues near Interface 2 to test the importance of the residues in oligomerization (Figure 2D). Five of the residues were on the SIRT2^cat^ C-terminal helix, and the sixth residue we mutated (Q142) dramatically repositioned to contact the C-terminal helix in myristoyl peptide-bound SIRT2^cat^, but not apo-SIRT2^cat^ (Figure 2C). Four of the point mutations had essentially no effect on SIRT2^cat^ dimerization in cross-linking experiments, whereas mutations Q142A and E340A reduced the dimeric population of enzyme from 47.4% for the wild-type protein to 25.4% (Q142A) or 40.1% (E340A) (Figure 2D and Table S2). Recombinant SIRT2^cat^ with the double mutation Q142A/E340A was 86.3% monomer after cross-linking which matched our observations of wild-type SIRT2 bound to myristoyl peptide (Figure 2D). We concluded that SIRT2^cat^ might dimerize in solution through Interface 2, or else the Q142A/E340A mutant had a structural alteration or conformational bias that affected dimerization. Not surprisingly, binding to the myristoyl peptide further stabilized the monomeric form of SIRT2^cat^(Q142A/E340A) (Figure 2E). Interestingly, binding to ADP-ribose promoted a dimeric form of SIRT2^cat^(Q142A/E340A) (Figure 2E). The mutant enzyme favored dimerization in the presence of ADP-ribose and acetyl peptide, but the mutant remained monomeric in the presence of the acetyl peptide alone (Figure 2F). Despite differences in the preferred oligomeric state between SIRT2^cat^ (dimer) and the Q142A/E340A mutant (monomer), our results confirmed the idea that substrate or small molecule binding could facilitate changes in SIRT2 oligomeric state and that the tendency to dimerize or form monomer is dependent on the identity of the substrate acyl chain.

Interestingly, Proteins, Interfaces, Structures, and Assemblies (PISA) software^38^ predicted that the biological assembly of apo-SIRT2^cat^ from PDB code 3ZGO was a homodimer that was not visible in the asymmetric unit, therefore providing another potential interface that could be responsible for SIRT2 dimerization (i.e., only half of the biological assembly predicted by PISA was in the asymmetric unit) (Figure 2G). The SIRT2^cat^ molecules in the PISA-predicted assembly were symmetrically oriented and had more extensive interactions than the interfaces in the asymmetric unit (Figure S7). Amino acid residues 286–313 were key residues at this predicted interface of the SIRT2^cat^ dimer (Figure 2G). We obtained a synthetic peptide of SIRT2 residues 286–313 to test whether this peptide disrupted SIRT2 dimer formation in cross-linking assays by competing for the predicted interface. This peptide had no effect on the ability of SIRT2^cat^ to form a dimer as indicated with cross-linking experiments (Figure 2H). This peptide also had no effect on the deacetylase or demyristoylase activities of SIRT2^cat^ when the peptide was added to reactions as high as 100 μM (Figure S8). It was possible that the 28mer peptide interacted at an interface but had substantially weaker affinity for SIRT2^cat^ than the protein had for itself, which would prevent the peptide’s ability to disrupt SIRT2^cat^ dimerization. However, the finding that high concentrations of the peptide (100 μM) had no effect on SIRT2^cat^ in our assays dampened our enthusiasm for pursuing this interface.

### SIRT2(Q142A/E340A) Has Normal Defatty-Acylase Activity and Impaired Deacetylase Activity

**Figure 3:**
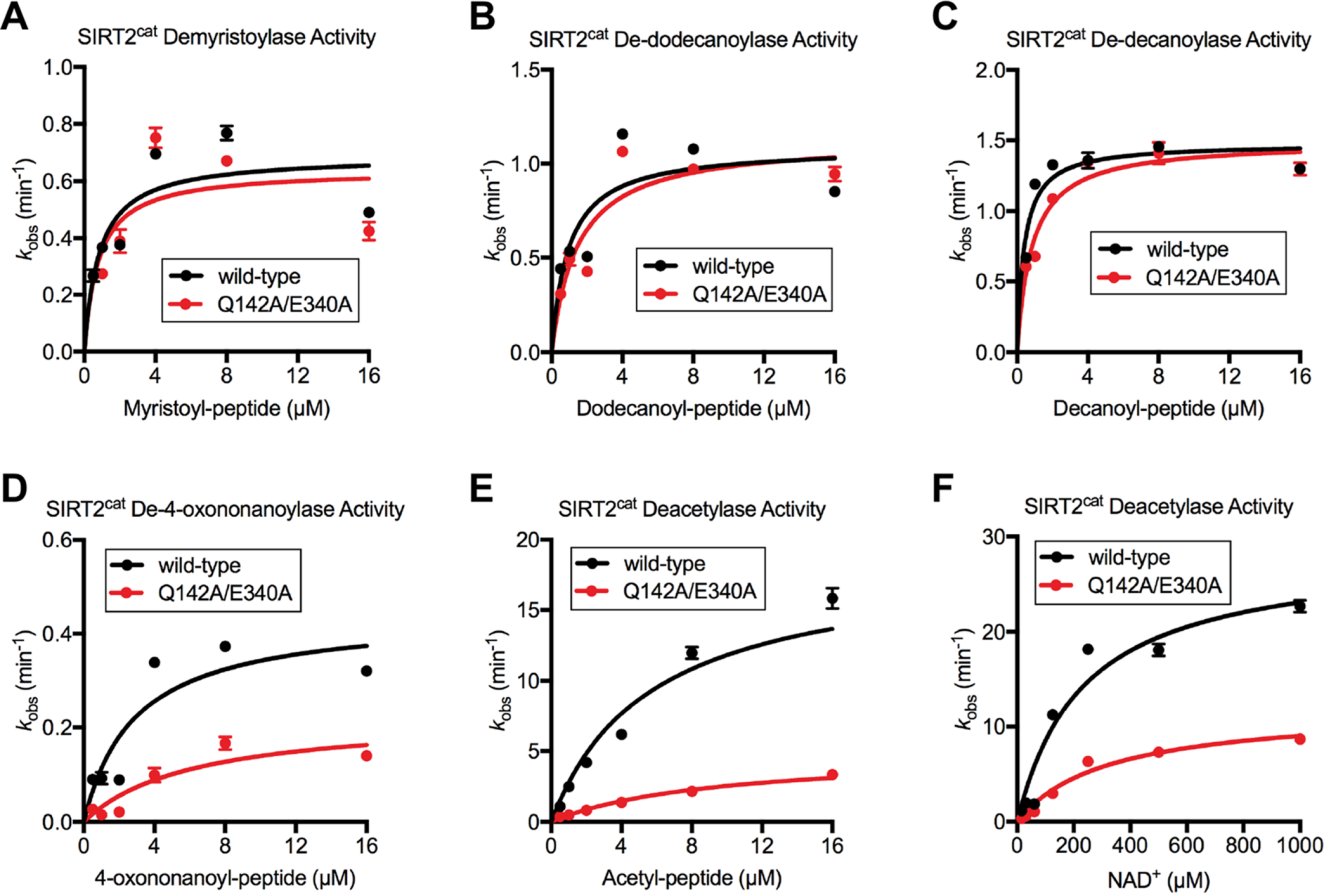
Dimerization-defective SIRT2^cat^(Q142A/E340A) has reduced deacetylase activity but normal demyristoylase activity. (A–E) Steady-state deacylase activities for SIRT2^cat^ and SIRT2^cat^(Q142A/E340A) performed with 1 mM NAD^+^ and varying concentrations of acylated peptides, as indicated in the panels. The mutant protein preparation from Figure 2E (lane 1) was used for these assays. (F) Steady-state deacetylase activity for SIRT2^cat^ and SIRT2^cat^(Q142A/E340A) performed with 16 μM acetyl peptide and varying concentrations of NAD^+^. Kinetic parameters *k*_cat_ and *K*_m_ for all kinetic curves can be found in Table 1.

We compared the steady-state deacylase activities of SIRT2^cat^ and SIRT2^cat^(Q142A/E340A) under saturating NAD^+^ conditions to determine how the mutations influence enzyme function. SIRT2^cat^ removes different acyl modifications with different efficiencies.^7,9^ The activity of SIRT2^cat^ and SIRT2^cat^(Q142A/E340A) were identical to each other in demyristoylase and dedodecanoylase assays (Figure 3A,B, and Table 1). The dedecanoylase activity of the two enzymes were very similar, with only a slight (∼2-fold) impairment of the mutant’s *K*_m_ (Figure 3C and Table 1). The activity of the mutant declined further relative to that of wild-type SIRT2^cat^ as the acyl chain was shortened. The catalytic efficiency (*k*_cat_/*K*_m_) for SIRT2^cat^(Q142A/E340A) was reduced 4-fold in de-4-oxononanoylase assays compared to SIRT2^cat^ (Figure 3D). The catalytic efficiency of the mutant was reduced 6-fold in deacetylase assays to wild-type SIRT2^cat^ (Figure 3E).

**Table 1:** Kinetic Parameters of SIRT2^cat^ and SIRT2^cat^(Q142A/E340A) for Acylated Peptides^a^

	SIRT2^cat^			SIRT2^cat^(Q142A/E340A)		
H4K16 peptide modification	*k*_cat_ (min^–1^)	*K*_m_ (μM)	*k*_cat_/*K*_m_ (μM^–1^ min^–1^)	*k*_cat_ (min^–1^)	*K*_m_ (μM)	*k*_cat_/*K*_m_ (μM^–1^ min^–1^)
acetyl	18.5 ± 1.0	5.7 ± 0.9	3.25	4.9 ± 0.2	9.4 ± 1.1	0.52
4-oxononanoyl	0.4 ± 0.1	2.9 ± 1.0	0.14	0.2 ± 0.1	6.2 ± 2.8	0.03
decanoyl	1.5 ± 0.1	0.4 ± 0.1	3.75	1.5 ± 0.1	0.8 ± 0.1	1.88
dodecanoyl	1.1 ± 0.1	0.9 ± 0.3	1.22	1.1 ± 0.1	1.5 ± 0.4	0.73
myristoyl	0.7 ± 0.1	0.8 ± 0.3	0.88	0.6 ± 0.1	0.8 ± 0.4	0.75
	*k*_cat, NAD+_ (min^–1^)	*K*_m, NAD+_ (μM)	*k*_cat_/*K*_m, NAD+_ (μM^–1^ min^–1^)	*k*_cat, NAD+_ (min^–1^)	*K*_m, NAD+_ (μM)	*k*_cat_/*K*m, NAD+ (μM^–1^ min^–1^)
acetyl	28.5 ± 2.4	234.1 ± 52.4	0.12	11.9 ± 0.8	317.5 ± 53.5	0.04

a Parameters for each of the five modifications were determined using 1 mM NAD^+^ (Figure 2A–E). Also determined were parameters for NAD^+^ using 16 μM acetyl peptide (Figure 2F). Standard error is shown.

We described above that SIRT2^cat^(Q142A/E340A) favored a monomeric form in cross-linking assays, but more readily formed a dimer in the presence of ADP-ribose (Figure 2E,F). However, ADP-ribose had no effect on the oligomeric state of wild-type SIRT2^cat^ (Figure S4). ADP-ribose and NAD^+^ bind the same site on SIRT2^cat^ in a similar orientation.^26,37^ Thus, it was plausible that the mutations altered the NAD^+^ site in a way that promoted binding of ADP-ribose and NAD^+^ to SIRT2^cat^(Q142A/E340A). To test the significance of this on enzyme function, we performed steady-state deacetylase assays under saturating acetyl peptide conditions (16 μM), and we varied the NAD^+^ concentration (Figure 3F). The *K*_m, NAD+_ for SIRT2^cat^ was 234 μM, which was similar to the value of 318 μM that we determined for SIRT2^cat^(Q142A/E340A). This indicated that the affinity of the mutant for NAD^+^ did not significantly change compared to wild-type SIRT2^cat^ during the deacetylase reaction. Interestingly, SIRT2^cat^ binds acylated substrate first during its reaction before interacting with NAD^+^.^12^ Whether this is also the case for SIRT2^cat^(Q142A/E340A), which binds ADP-ribose in the absence of an acylated substrate (Figure 2E,F), requires further study.

### SIRT2 Can Dimerize in Human Cells

**Figure 4:**
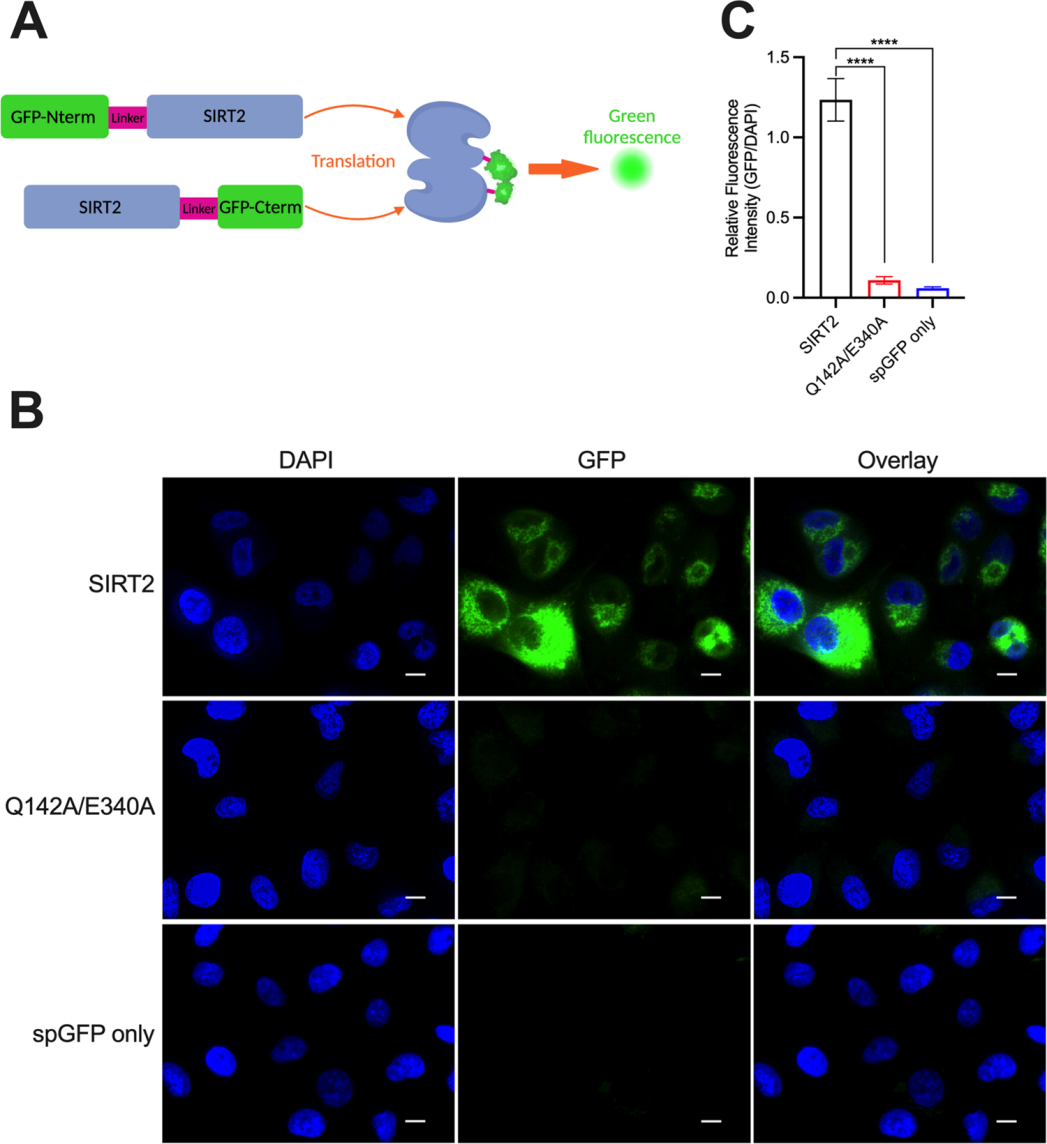
Detection of SIRT2 dimerization in cells. (A) Experimental scheme where full-length SIRT2 was tagged with an N-terminal split GFP fragment and a separate SIRT2-fusion protein contained the complementary C-terminal split GFP fragment. If SIRT2 dimerizes in cells, the two split GFP fragments come together and fluoresce. (B) The top row shows fluorescence imaging of A549 cells expressing the split GFP fragment-tagged SIRT2 proteins 48 h after transfection. The overlay shows GFP fluorescence in the cytosol, indicating that SIRT2 dimerized in that compartment, but not the DAPI-stained nucleus. In the middle row of images, an identical experiment was performed using split GFP fragment-tagged SIRT2(Q142A/E340A) proteins, which did not yield GFP fluorescence because the mutant was defective in its ability to dimerize. In the bottom row of images, the split GFP fragments were simultaneously expressed without being fused to a SIRT2 protein. The scale bar is 10 μm. (C) Quantification of the GFP fluorescence intensities from the images shown in (B). The GFP fluorescence intensities from individual cells were normalized to the DAPI fluorescence intensities from the same cells. The relative fluorescence intensities were compared with a one-way ANOVA followed by Dunnett’s multiple comparison test (****p < 0.0001).

We examined the potential for full-length SIRT2 to dimerize in human lung cancer cells (A549) using a split-GFP approach.^28^ We simultaneously expressed two SIRT2 constructs, where one form contained a GFP fragment on its N-terminus, and the other SIRT2 construct contained the complementary GFP fragment on its C-terminus (Figure 4A). Fluorescence would result if the two SIRT2 proteins dimerized in cells and the split GFP fragments came together.^28^ Indeed, the GFP signal was observed in the cytosol when the two SIRT2 constructs were simultaneously expressed, indicating that the proteins dimerized in cells (Figures 4B and S9). As a negative control, we performed an identical experiment where we expressed dimerization-defective SIRT2(Q142A/E340A) mutants that each contained a GFP fragment. In the A549 cells, the GFP signal was significantly reduced when the split GFP fragments were fused to SIRT2(Q142A/E340A) compared to wild-type SIRT2 (Figures 4B,C and S9), indicating a lack of dimerization that was consistent with cross-linking experiments (Figure 2D–2F). As an additional negative control, we simultaneously expressed both split GFP fragments without them being fused to SIRT2 proteins. This condition also yielded weak fluorescence, further validating that SIRT2 dimerization in cells was essential to bring the split GFP fragments together (Figure 4B,C).

### Pharmacologic Dissociation of SIRT2 Dimers by a Deacetylase and Defatty-Acylase Inhibitor

**Figure 5:**
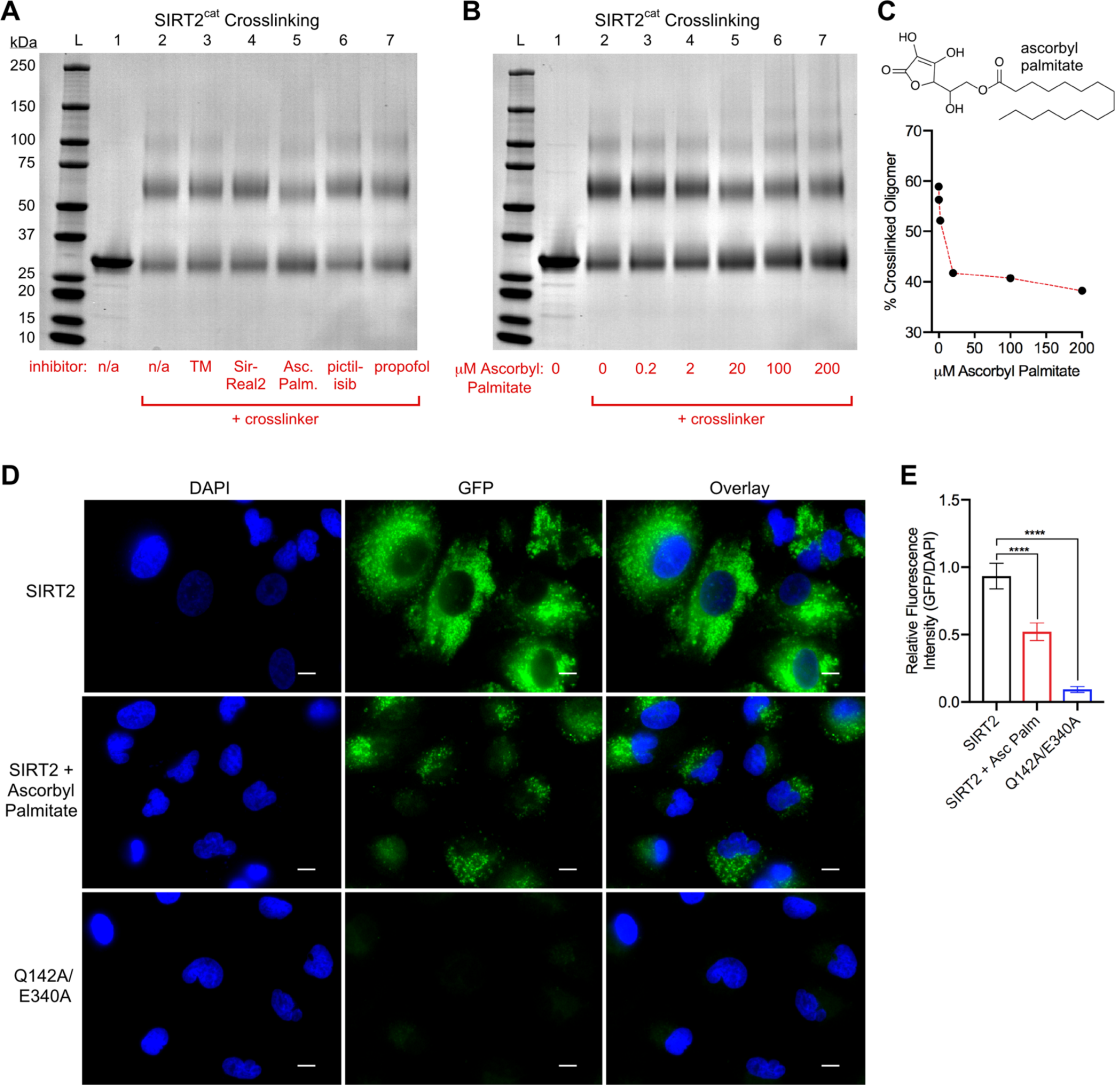
Reduction of SIRT2 oligomerization by ascorbyl palmitate. (A) Cross-linking of 10 μM SIRT2^cat^ with Bis(NHS)-PEG5 as visualized by SDS-PAGE. Cross-linker was omitted from lane 1, and additional components for each lane include: 2, SIRT2^cat^ alone; 3, 12 μM TM; 4, 12 μM SirReal2; 5, 20 μM ascorbyl palmitate; 6, 30 μM pictilisib; 7, 200 μM propofol. See the Materials and Methods section for justification of ligand concentrations. (B) 10 μM SIRT2^cat^ cross-linking with Bis(NHS)-PEG5 in the presence of the indicated concentrations of ascorbyl palmitate. (C) Quantification of the percent of SIRT2^cat^ that cross-linked as an oligomer (dimer or trimer) under different ascorbyl palmitate concentrations from the gel in (B). The chemical structure of ascorbyl palmitate is also shown. (D) Fluorescence imaging of A549 cells expressing split GFP fragment-tagged SIRT2 proteins, as in Figure 4. 24 h after transfection, the cells were given fresh media for 6 h containing 200 μM ascorbyl palmitate and 0.2% DMSO (middle row of images) or 0.2% DMSO alone (top and bottom rows). The scale bar is 10 μm. (E) Quantification of the GFP fluorescence intensities from the images shown in (D). The GFP fluorescence intensities from individual cells were normalized to the DAPI fluorescence intensities from the same cells. The relative fluorescence intensities were compared with a one-way ANOVA followed by Dunnett’s multiple comparison test (****p < 0.0001).

To test whether SIRT2 dimerization could be pharmacologically disrupted, we cross-linked 10 μM SIRT2^cat^ as before, but pre-equilibrated the protein with various inhibitors at concentrations that are sufficient to saturate the enzyme. TM and SirReal2 are SIRT2 deacetylase inhibitors with nM potency *in vitro*(4,15,26) that had no effect on SIRT2^cat^ dimerization (Figure 5A, lanes 3 and 4). Pictilisib and propofol inhibit various SIRT2 deacylase activities with μM potency,^9,14^ but also had no effect on SIRT2^cat^ dimerization (Figure 5A, lanes 6 and 7). Interestingly, ascorbyl palmitate is another inhibitor of SIRT2 deacetylase and defatty-acylase activities with low μM potency,^14^ and it reduced the amount of cross-linked SIRT2^cat^ dimer from 49.9 to 32.1% (Figure 5A, lane 5, and Table S2). We confirmed this result by cross-linking SIRT2^cat^ in the presence of a wider range of ascorbyl palmitate concentrations (Figure 5B,C). The dissociation of the enzyme oligomer by ascorbyl palmitate was consistent but incomplete; in this experiment, 31.8% of the enzyme remained a dimer in the presence of 200 μM ascorbyl palmitate compared to 50.6% of SIRT2^cat^ in the absence of inhibitor (Figure 5B,C, and Table S2). Nonetheless, we examined whether ascorbyl palmitate could disrupt SIRT2 dimerization in cells using our split-GFP system. Ascorbyl palmitate inhibits SIRT2 deacetylase and defatty-acylase activities in cells, but its potency is reduced ∼10-fold compared to its inhibition of SIRT2 in purified enzyme assays.^14^ Regardless, the dose range of ascorbyl palmitate that affects SIRT2 activity in cells (150–200 μM)^14^ significantly reduced SIRT2 dimerization in cells (Figure 5D,E).

## Discussion

**Figure 6:**
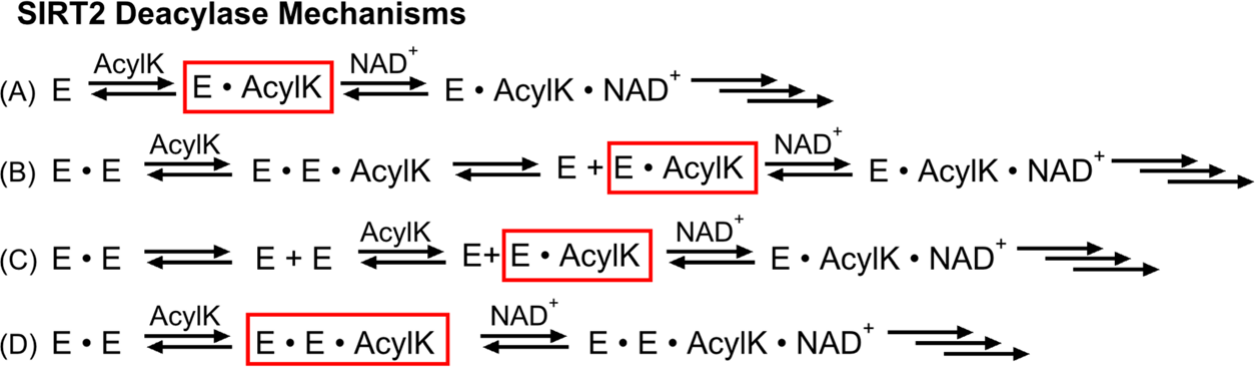
Proposed deacylation mechanisms for SIRT2. (A) SIRT2 reaction mechanism as previously described.^12^ Monomeric SIRT2 binds acylated lysine substrate (AcylK) followed by NAD^+^, and then the reaction proceeds. This mechanism would occur for all SIRT2 deacylase reactions when the enzyme concentration is low and SIRT2 is monomeric. For all panels, the red boxed complex can bind NAD^+^. (B) Proposed mechanism for SIRT2 long fatty deacylase reactions where an enzyme dimer binds the acylated substrate, and a resulting conformational change dissociates the enzyme into monomers. The SIRT2 monomer bound to the acylated substrate can then bind NAD^+^. This mechanism would occur when SIRT2 concentrations favor dimer formation and would be similar for any acyl substrate that dissociates SIRT2 dimers into monomers. (C) Additional possible mechanism for SIRT2 long fatty deacylase reactions at high enzyme concentrations where an enzyme dimer must dissociate into monomers first, then the monomers can bind acylated substrate. Mechanisms B and C are not mutually exclusive. (D) Proposed mechanism for SIRT2 short-chain deacylase (e.g., deacetylase) reactions at high SIRT2 concentrations where the dimer is favored and the enzyme does not dissociate into monomers upon substrate binding. Dimerization enhances the reaction kinetics, and this mechanism would apply to any acyl substrate that binds a SIRT2 dimer without dissociating the dimer.

This work demonstrated that human SIRT2 is capable of dimerizing in solution and in cells, that the oligomeric state of SIRT2 can be changed by interactions with specific substrates or small molecules, and that the dimerization of SIRT2 differentially influences its deacylase activities. Monomeric and dimeric SIRT2 exist at an equilibrium that is sensitive to perturbations in the active site, for example, the enzyme is compelled to adopt a monomeric state when bound to long fatty acylated substrates such as decanoyl or myristoyl modifications (Figure 1D). Mutations that promote the monomeric state of SIRT2 have no effect on SIRT2’s long fatty deacylase activities (Figure 3 and Table 1). In contrast, we have not observed a dimer-to-monomer transition induced by the acetyl substrate (Figure 1D), and mutations that disrupt dimerization selectively reduce SIRT2’s deacetylase and short-chain deacylase activities (Figure 3 and Table 1). The SIRT2 mutations (Q142A/E340A) that disrupted dimer formation might be located at the dimer interface, but alternative dimeric species are predicted to exist with alternative interfaces (Figure 2G and Table S1), and it is possible that these mutations induced a conformational bias that selectively weakened the deacetylase and de-4-oxonanonoylase activities of the SIRT2 monomer. The implications of our findings for SIRT2’s reaction mechanism are summarized in Figure 6.

An important aspect to consider is the concentration dependence of SIRT2 dimerization in the absence of substrate. We caution that cross-linking may not report the actual fraction of monomer and dimer in solution because its efficiency is dependent on the concentration of macromolecule in solution regardless of whether the macromolecule has concentration-dependent oligomeric states, and the cross-linking chemistry was not specific to any dimer interface. However, the analytical SEC experiments did report actual concentrations of monomer and dimer at equilibrium as the protein exited the column by virtue of the UV–vis absorption measurements of the peaks (Figure 1A). We used these data to estimate a *K*_d_ of 121 ± 12 nM for the self-association of SIRT2^cat^ (mean ± S.D.) (see the Materials and Methods section). Proteomic experiments estimated that the concentration of SIRT2 in mammalian cell lines was in a similar range at approximately 30–100 nM (see the Materials and Methods section).^29^ Furthermore, SIRT2 comprises an astounding ∼1% of the total protein content in myelin where the cytosolic volume is naturally reduced.^39−41^ Thus, it is likely that some fraction of SIRT2 can dimerize in cells under native conditions, and this may change with the level of protein expression. We note that our enzyme activity assays with wild-type SIRT2^cat^ were performed under conditions where a mixture of monomeric and dimeric enzyme was likely present, and we could not precisely quantify the full effect of dimerization on reaction rate (see the Materials and Methods section). We cross-linked SIRT2 at very low concentrations to measure the presence of monomer and dimer during enzyme reactions, but this was beneath the detection limit for Coomassie staining, and the ability of our antibody to recognize SIRT2 was impeded by the cross-linking reagents (both NHS ester-based and formaldehyde).

The ability of SIRT2 to dimerize appears to be linked to its proper functioning as a deacetylase but not as a long fatty deacylase. SIRT2 dimers also have distinct surfaces available for drug targeting compared to SIRT2 monomers (Table S1), which may allow for selective targeting of oligomeric states to influence its different deacylase activities. We found that the SIRT2 deacetylase and defatty-acylase inhibitor ascorbyl palmitate shifted SIRT2’s equilibrium toward a monomeric state (Figure 5). Interestingly, ascorbyl palmitate resembles the long fatty acyl substrates that also promote the monomeric state (Figure 5C), but whether its mechanism of SIRT2 inhibition is related to its effects on SIRT2 dimerization is not yet clear. We note that TM is a much more potent inhibitor of SIRT2 and is a thiomyristoyl-containing, mechanism-based inhibitor that also resembles long fatty acyl substrates, yet it did not promote SIRT2 monomerization (Figure 5A).

Altogether, we conclude that investigating the basis for SIRT2 oligomerization, the effects of small molecules on oligomerization, and the effects of oligomerization on its activity are critical for understanding the regulation of this deacylase enzyme in many biological processes. With continued improvements in genetic engineering technologies, we also postulate that engineered mutants such as SIRT2(Q142A/E340A) have the potential to clarify the role of different SIRT2 activities in normal and diseased cells.

## Supporting Information Available

Predicted and observed oligomeric states (Table S1); quantification of SIRT2 oligomeric states (Table S2); X-ray crystallography data collection and refinement statistics (Table S3); interactions between SIRT2^cat^ molecules in the asymmetric unit of PDB code 3ZGO (Table S4); SEC trace (Figure S1); Coomassie-stained SDS-PAGE gel (Figure S2); interaction of SIRT2^cat^ with acetyl-H4K16 peptide (Figure S3); Coomassie-stained SDS-PAGE gel (Figure S4); interactions of SIRT2^cat^ with FAM-myristoyl-H4K16 peptide (Figure S5); SEC chromatogram (Figure S6); PISA software output (Figure S7); synthetic peptide containing residues 286-313 of SIRT2 had no effect on the deacetylase or demyristoylase activities of SIRT2^cat^ (Figure S8); and detection of SIRT2 dimerization in cells (Figure S9) (PDF)

## Accession Codes

SIRT2, Q8IXJ6
