# Supplementary material for: Effects of Dimerization on the Deacylase Activities of Human SIRT2
Source: Biochemistry. 2023 Nov 15;62(23):3383–95. doi: 10.1021/acs.biochem.3c00381 (PMC10702427; doi:10.1021/acs.biochem.3c00381)
Supplement: Supplementary file 1 — bi3c00381_si_001.pdf [file bi3c00381_si_001.pdf]

Supporting Information

Effects of Dimerization on the Deacylase Activities of Human SIRT2

Jie Yang,[a] Nathan I. Nicely,[b] Brian P. Weiser*[a]

[a]Department of Molecular Biology, Rowan University School of Osteopathic Medicine, Stratford, NJ 08084

[b]Department of Pharmacology, University of North Carolina at Chapel Hill, Chapel Hill, NC 27599

*corresponding author:

Brian P. Weiser

2 Medical Center Dr.

Stratford, NJ 08084

Phone: (856) 566-6270

Email: weiser@rowan.edu

Table S1. Predicted and Observed Oligomeric States of SIRT2^{cat} from 38 X-ray Crystal Structures

PDB code	SIRT2 molecules in asymmetric unit	Ligands or substrate bound to SIRT2[A]	Top ranked PISA-predicted biological assembly [1]	Predicted biological assembly in asymmetric unit?	Notes	Reference
1J8F	3	N/A	dimer	No	original apo-SIRT2 structure before re-refinement (see 3ZGO)	2
3ZGV	2	ADP-ribose	dimer[B]	Yes	"pseudo-substrate" bound protein; active site occupied by Leu297 from adjacent molecule	3
3ZGO	3	N/A	dimer	No	re-refined structure of 1J8F	3
4L3O	4	macrocyclic peptide inhibitor S2iL5	dimer	No		4
4RMG	1	SirReal2 inhibitor and NAD^+	dimer[C]	No		5
4RMH	1	SirReal2 inhibitor and acetyl-peptide	monomer	Yes		5
4RMI	1	SirReal1 inhibitor and acetyl-peptide	monomer	Yes		5
4RMJ	2	ADP-ribose and nicotinamide	dimer[B]	Yes	"pseudo-substrate" bound protein; active site occupied by Leu297 from adjacent molecule	5
4R8M	2	thiomyristoyl-peptide	monomer	No		6
5D7O	2	ADP-ribose	dimer[B]	Yes	"pseudo-substrate" bound protein; active site occupied by Leu297 from adjacent molecule	7
5D7P	2	ADP-ribose and inhibitor EX-243	dimer[B]	Yes	"pseudo-substrate" bound protein; active site occupied by Leu297 from adjacent molecule	7
5D7Q	2	ADP-ribose and inhibitor CHIC35	dimer[B]	Yes	"pseudo-substrate" bound protein; active	7

					site occupied by Leu297 from adjacent molecule	
4Y6L	2	myristoyl-peptide	monomer	No		[8]
4Y6O	2	myristoyl-peptide	monomer	No		[8]
4Y6Q	4	2′-*O*-myristoyl-ADP-ribose	monomer	No		[8]
4X3O	1	thiomyristoyl-peptide and NAD^+ (reaction intermediate)	monomer	Yes		[9]
4X3P	1	myristoyl-peptide and Carba-NAD^+	monomer	Yes		[9]
5DY4	1	SirReal inhibitor analog and NAD^+	dimer[C]	No		[10]
5DY5	1	SirReal inhibitor analog	dimer[C]	No		[11]
5FYQ	2	trifluoroacetyl-peptide	monomer	No		[12]
5MAT	2	thienopyrimidinone inhibitor	dimer	No	The predicted biological assembly (dimer) is different than the dimer in the asymmetric unit	[13]
5MAR	2	ADP-ribose and 1,2,4-oxadiazole inhibitor	dimer[B]	Yes	“pseudo-substrate” bound protein; active site occupied by Leu297 from adjacent molecule	[14]
5G4C	2	4-oxononanoyl peptide and Carba-NAD^+	monomer	No		[15]
5Y5N	1	2-anilinobenzamide inhibitor	monomer	Yes		[16]
5Y0Z	2	inhibitor NPD11033	monomer	No		[17]
5YQL	1	inhibitor A2I	monomer	Yes		[18]
5YQM	1	inhibitor A29	monomer	Yes		[18]
5YQN	1	inhibitor L55	monomer	Yes		[18]
5YQO	1	inhibitor L5C	monomer	Yes		[18]
6QCN	2	ADP-ribose and quercetin	dimer[B]	Yes	“pseudo-substrate” bound protein; active site occupied by Leu297 from adjacent molecule	[19]
6NR0	2	peptide-like thiomyristoyl inhibitor and NAD^+ (reaction intermediate)	dimer	Yes		[20]

6L65	1	myristoyl-peptide	monomer	Yes		Chen, LF PDB DOI: 10.2210/pdb6L65/pdb
6L66	1	thiomyristoyl-peptide and NAD^+	monomer	Yes		Chen, LF PDB DOI: 10.2210/pdb6L66/pdb
7BOS	1	peptide-like thiomyristoyl inhibitor	monomer	Yes		[21]
7BOT	1	peptide-like thiomyristoyl inhibitor	monomer	Yes		[21]
6L71	1	myristoyl-peptide and NAD^+ (reaction intermediate)	monomer	Yes		Chen, LF PDB DOI: 10.2210/pdb6L71/pdb
6L72	1	2′-*O*-myristoyl-ADP-ribose	monomer	Yes		Chen, LF PDB DOI: 10.2210/pdb6L72/pdb
8TGP	1	myristoyl-peptide	monomer	Yes		Current work

[A]Common salts and chemicals from crystallization screening buffers were omitted.
[B]These biological assemblies are the same.
[C]These biological assemblies are the same.

Figure S1. Representative size exclusion chromatography (SEC) trace from the purification of SIRT2^{cat} from *E. coli* (top), and a Coomassie-stained SDS-PAGE gel of various fractions surrounding the chromatography peak (bottom). Notably, SIRT2^{cat} elutes as one broad peak during this preparatory chromatography, which contrasts the two peaks observed when analytical amounts of SIRT2^{cat} are analyzed with SEC (see Figure 1 of the main article).

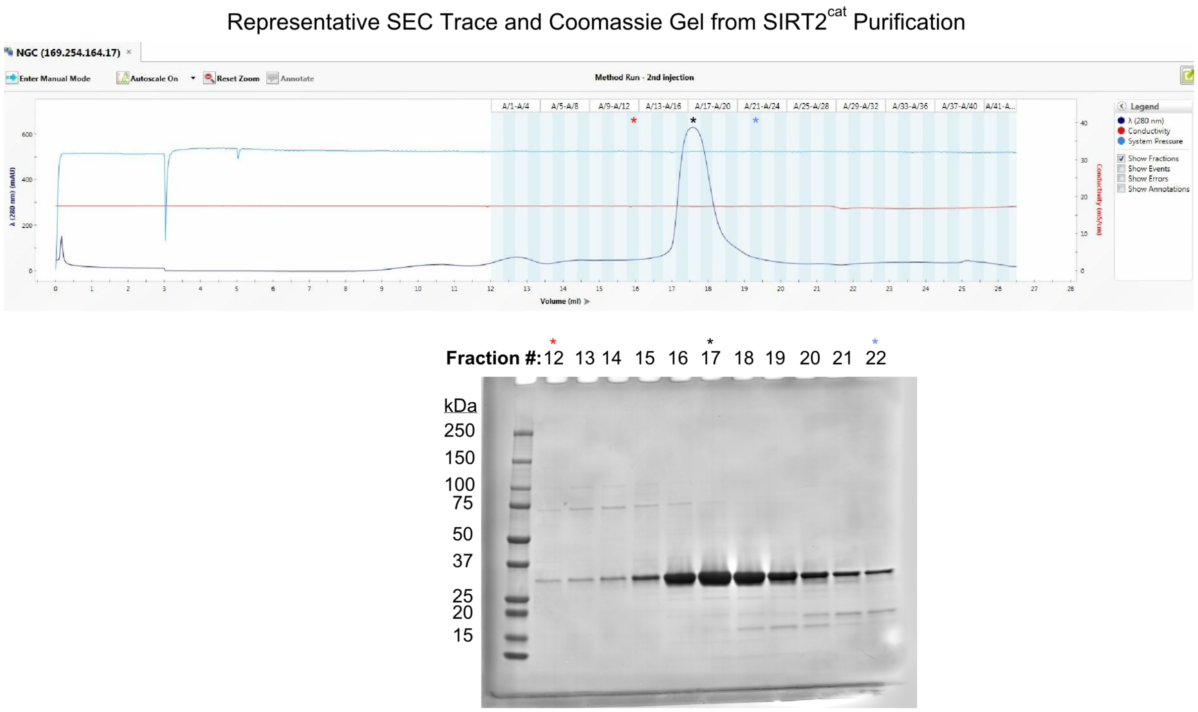

Figure S2. Coomassie-stained SDS-PAGE gel showing a crosslinking experiment with 10 µM SIRT2^{cat} and the indicated amounts of formaldehyde. Monomeric, dimeric, and (to a lesser extent) trimeric forms of SIRT2^{cat} were visible as they were with the NHS-ester based crosslinker used in the main article.

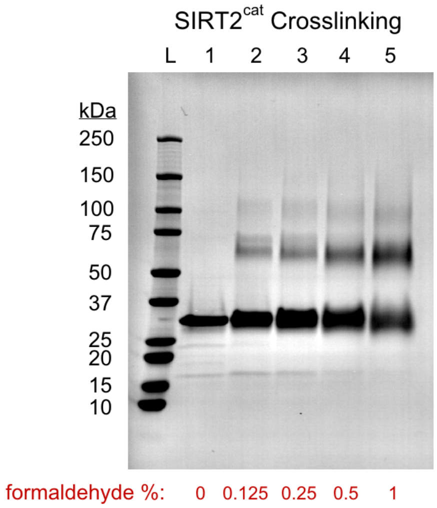

Table S2. Quantification of SIRT2 Oligomeric States from All Reported Crosslinking Gels

Figure	Oligomeric States	Lane 1	Lane 2	Lane 3	Lane 4	Lane 5	Lane 6	Lane 7	Lane 8
Figure 1B	% Monomer	100.0	100.0	43.2	84.0	87.0	40.1		
	% Dimer	0.0	0.0	50.8	16.0	13.0	52.5		
	% Trimer	0.0	0.0	6.0	0.0	0.0	7.4		
Figure 1D	% Monomer	100.0	100.0	50.6	57.0	61.6	92.3	90.9	93.7
	% Dimer	0.0	0.0	48.2	41.7	37.1	7.7	9.1	6.3
	% Trimer	0.0	0.0	1.2	1.3	1.3	0.0	0.0	0.0
Figure 2D (left panel)	% Monomer	39.3	72.9	45.3	59.9	50.2	39.0		
	% Dimer	47.4	25.4	50.5	40.1	47.8	58.8		
	% Trimer	13.3	1.7	4.2	0.0	2.0	2.2		
Figure 2D (right panel)	% Monomer	35.9	38.1	86.3					
	% Dimer	57.4	55.6	13.7					
	% Trimer	6.7	6.3	0.0					
Figure 2E	% Monomer	100.0	87.2	100.0	54.1	100.0			
	% Dimer	0.0	12.8	0.0	45.9	0.0			
	% Trimer	0.0	0.0	0.0	0.0	0.0			
Figure 2F	% Monomer	100.0	77.2	79.5	45.7	47.8			
	% Dimer	0.0	22.8	20.5	53.5	51.3			
	% Trimer	0.0	0.0	0.0	0.8	0.9			
Figure 2H	% Monomer	100.0	60.3	58.7	59.5	63.2	62.6		
	% Dimer	0.0	36.0	36.3	37.5	33.2	34.9		
	% Trimer	0.0	3.7	5.0	3.0	3.6	2.5		
Figure 5A	% Monomer	100.0	45.6	43.2	48.4	66.1	43.2	53.3	
	% Dimer	0.0	49.9	51.3	51.6	32.1	52.1	43.1	
	% Trimer	0.0	4.5	5.5	0.0	1.8	4.7	3.6	
Figure 5B	% Monomer	100.0	41.1	43.7	47.9	58.3	59.3	61.8	
	% Dimer	0.0	50.6	48.8	45.9	37.3	33.1	31.8	
	% Trimer	0.0	8.3	7.5	6.2	4.4	7.6	6.4	
Figure S2	% Monomer	100.0	82.0	81.4	73.6	59.1			
	% Dimer	0.0	16.2	16.3	23.9	37.7			
	% Trimer	0.0	1.8	2.3	2.5	3.2			
Figure S4	% Monomer	100.0	33.0	37.7	30.7	36.1			
	% Dimer	0.0	58.2	55.4	58.6	54.8			
	% Trimer	0.0	8.8	6.9	10.7	9.1			

Figure S3. Interaction of SIRT2^{cat} with acetyl-H4K16 peptide. Note that similar experiments were reported in a previous work[22] and were repeated for this manuscript. (A) Chemical structure of Cy3-myristoyl-H4K16 peptide. The internal lysine was myristoylated, and a PEG4 linker was attached to the N-terminal amine of the peptide as opposed to the lysine side chain. (B) The affinity of SIRT2^{cat} for Cy3-myristoyl-H4K16 peptide was determined by measuring changes in Cy3 fluorescence emission in the presence of increasing SIRT2^{cat} concentrations. (C) Binding competition assays where Cy3-myristoyl-H4K16 peptide was competed off 2.5 μM of SIRT2^{cat} by increasing amounts of unlabeled acetyl-H4K16 peptide and unlabeled myristoyl-H4K16 peptide. Relevant to main text of the article, the inhibition data for acetyl-H4K16 peptide determined that 12 μM of peptide reduced Cy3-myristoyl-H4K16 peptide binding to SIRT2^{cat} by ~25% which demonstrates acetyl peptide binding to SIRT2^{cat}.

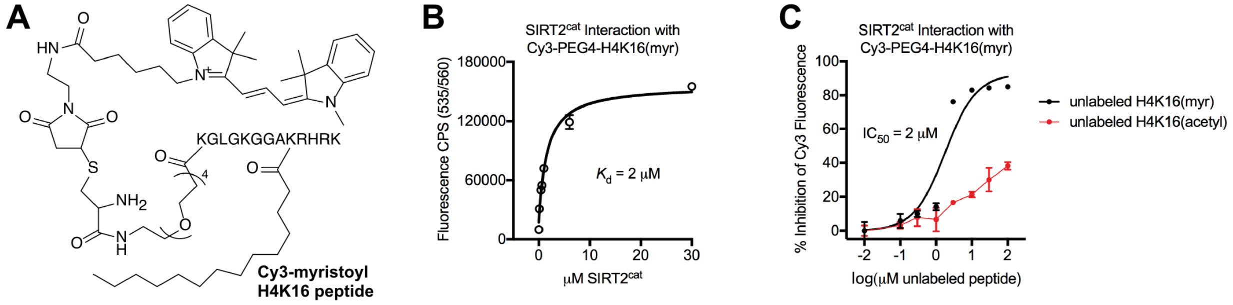

Figure S4. Coomassie-stained SDS-PAGE gel showing a crosslinking experiment with 10 μM SIRT2^{cat} and Bis-(NHS)-PEG5 as the crosslinker; crosslinker was omitted from lane 1. When included, the concentration of acetyl peptide substrate was 12 μM and the concentration of ADP-ribose was 300 μM.

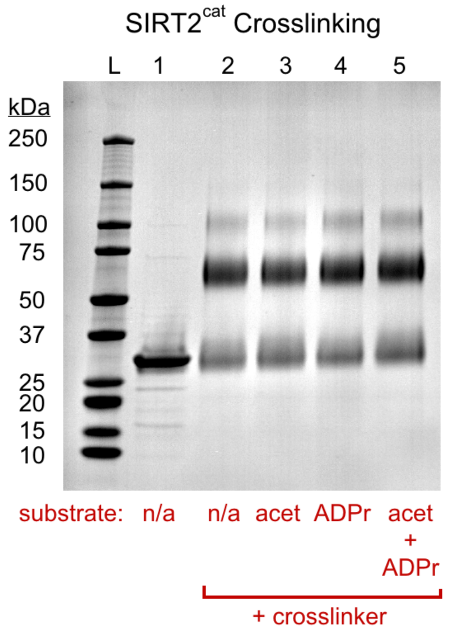

Figure S5. Interactions of SIRT2^{cat} with FAM-myristoyl-H4K16 peptide. (A) Native PAGE EMSA gel where 2 μM SIRT2^{cat} was equilibrated with the indicated myristoyl peptide concentrations prior to electrophoresis. The gel shift occurs when SIRT2^{cat} binds peptide because the -8 charge of SIRT2^{cat} becomes reduced when bound to the basic peptides, which had a charge of +5, thus slowing migration of the protein/peptide complex towards the positive electrode. The fluorescence image was taken prior to staining with Coomassie blue and re-imaging. (B) Affinity of SIRT2^{cat} for myristoyl-H4K16 peptide and FAM-myristoyl-H4K16 peptide as determined by quantifying the fraction of SIRT2^{cat} that gel shifted upon peptide binding in native PAGE EMSA. (C) Demyristoylation of FAM-myristoyl-H4K16 peptide by SIRT2^{cat} determined with MALDI-MS. 1 μM of the peptide was reacted with 0.2 μM SIRT2^{cat} and 1 mM NAD^+ for 30 minutes at 37°C. The 210 Da shift corresponds to the loss of the myristoyl modification on the peptide. For reference, the calculated average mass of the peptide is 2209 Da, and thus the +1 ions were detected on the spectra. (D) Representation of the crystal structure of SIRT2^{cat} bound to FAM-myristoyl-H4K16 peptide; only five amino acids of the substrate peptide were resolved in the structure, as is common for sirtuin structures. SIRT2^{cat} is colored magenta, and the most N-terminal peptide residue visible in the structure was Gly14, which is colored by atom with carbon being yellow. The myristoyl modification is colored green, and the rest of the peptide backbone is colored by atom with carbon being cyan. The N-terminal portion of the peptide containing FAM was likely disordered and not resolved in the structure. X-ray crystallography methods and statistics can be found in the Table S3.

(Figure S5)

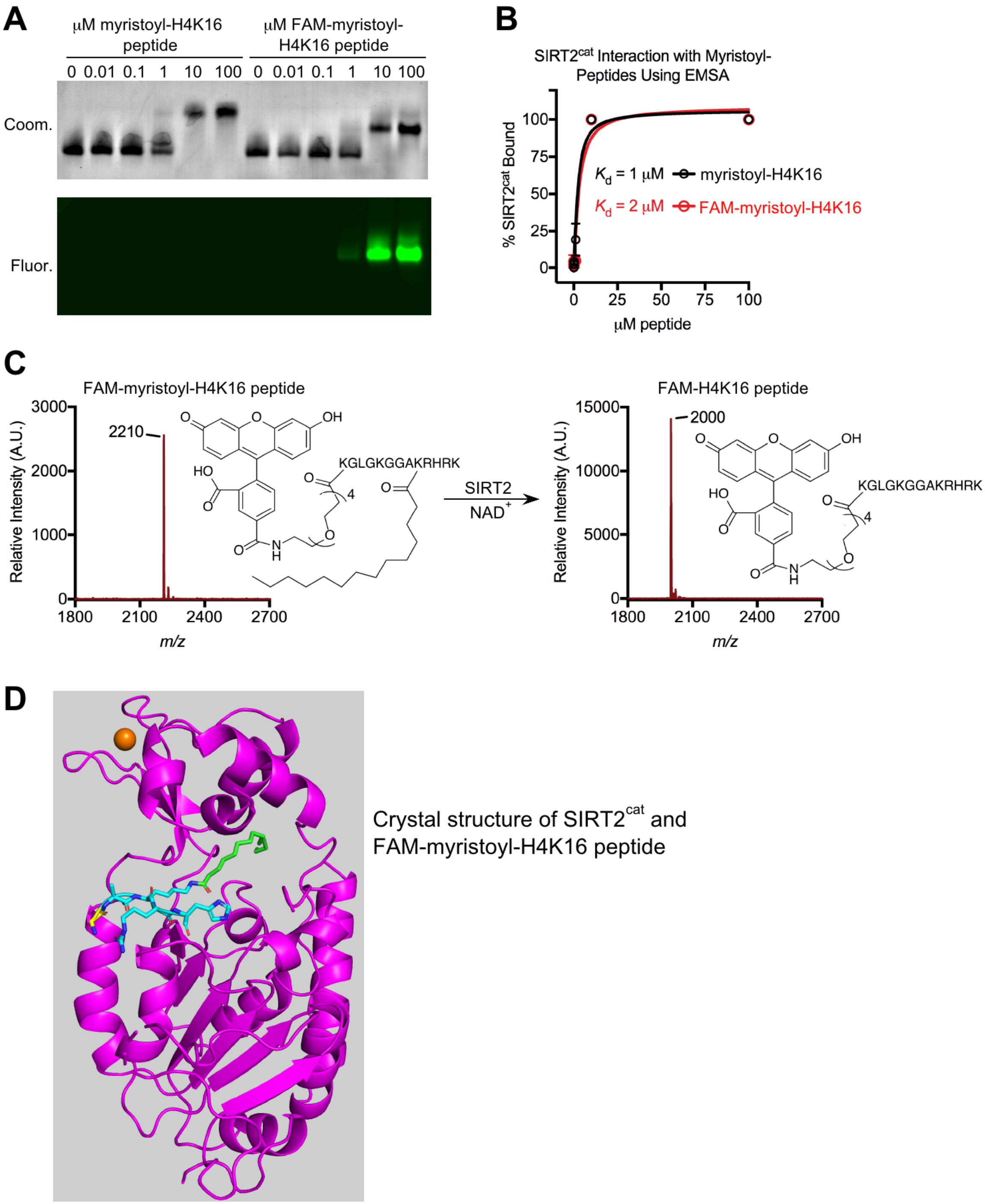

Crystal structure of SIRT2^{cat} and FAM-myristoyl-H4K16 peptide

Table S3. X-ray Crystallography Data Collection and Refinement Statistics

**Data reduction**	
Wavelength (Å)	1.0
Resolution range[A]	50.00-1.76 (1.79-1.76)
Space group	P 1 21 1
Unit cell	37.138 (a, Å)
	76.995 (b, Å)
	56.005 (c, Å)
	90 (α, deg)
	97.894 (β, deg)
	90 (γ, deg)
Total reflections	96339
Unique reflections	29902 (1339)
Multiplicity	3.2 (3.0)
Completeness (%)	96.0 (86.4)
Mean I/sigma (I)	25.5 (2.6)
R-merge	0.032 (0.328)
R-pim	0.023 (0.254)
CC1/2	0.996 (0.923)
CC*	0.999 (0.980)
**Model refinement**	
Total reflections used	27073 (914)
R-work	0.1902 (0.2926)
R-free	0.2304 (0.3550)
Non-hydrogen atoms	2340
Macromolecules[B]	2243
Ligands[B]	15
Solvent	83
Ramachandran favored (%)	97.44
Ramachandran allowed (%)	2.56
Ramachandran outliers	0
Rotamer outliers	0.00
Clashscore	4.67
Avg. B-factor, macromolecules	28.62
Avg. B-factor, ligands	35.13
Avg. B-factor, solvent	26.17
RMSD bonds	0.009
RMSD angles	1.000

[A]Parentheses denote figures for the highest resolution shell.

[B]Synthetic peptide amino acid residues are included in the ‘macromolecule’ count; only atoms from the myristoyl group (on the modified Lys residue) are included in the ‘ligands’ count.

Figure S6. Analytical SEC chromatogram of full-length SIRT2 showing primary elution of dimeric protein at 68 kDa, and a smaller monomer shoulder peak at 43 kDa.

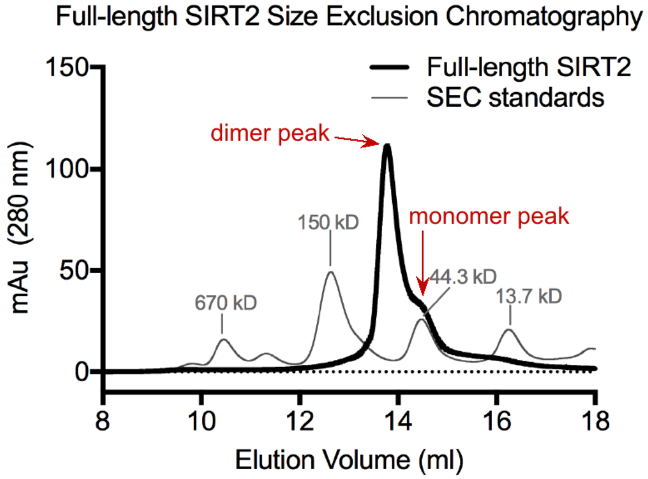

Table S4. Interactions Between SIRT2^{cat} Molecules in the Asymmetric Unit of PDB Code 3ZGO

**Interface 1 (defined in main text)**			
**Chain A[A]**	**Chain B**	**Minimum distance**	**Nature of contact**
Arg57	Glu74	3.2 Å	side chain–side chain salt bridge
Asp60	Arg75	3.2 Å	side chain–backbone hydrogen bond
Glu65	Asp60	3.3 Å	side chain–side chain interaction (C–O contact)
Glu61	Ser73	3.3 Å	side chain – side chain interaction (C–O contact)
Arg69	Leu58	3.7 Å	side chain–backbone hydrogen bond
**Interface 2 (defined in main text)**			
**Chain C**	**Chain B**	**Minimum distance**	**Nature of contact**
Arg347	Arg346	3.5 Å	side chain–side chain hydrogen bond
Glu340	Arg347	3.6 Å	side chain–side chain interaction (C–O contact)
Lys339	Ala350	4 Å	side chain–backbone hydrogen bond

[A]Chains A, B, and C represent individual SIRT2 molecules in the asymmetric unit of the crystal structure. These are labeled to be consistent with their chain ID in the crystal structure.

Figure S7. PISA software[1] output from analysis of apo-SIRT2^{cat} crystal structure (PDB code 3ZGO). The highest scored, predicted oligomeric state was a protein dimer shown in Figure 2G of the main article. For the predicted SIRT2^{cat} dimer (as shown below), protein-protein interactions included twenty hydrogen bonds, seven of which were categorized as salt bridges, and a large buried surface area at the interface (6210 Å^2 buried area compared to 27787 Å^2 of total solvent-accessible surface). Also related to Figure 2G and 2H in the main article, as well as Figure S8: For each SIRT2^{cat} molecule in the predicted dimer, twenty-six of the twenty-eight residues from amino acids 286-313 made contacts with the other SIRT2^{cat} molecule including four hydrogen bonds (two of which were salt bridges).

PISA Assembly.

Session Map (id=717-NM-626)

Start | Warnings | Interfaces | Interface Search

Monomers

Assemblies

Probable Assembly [1] in PDB 3zgo crystal.

Space symmetry group: C 2 2 21. Resolution: 1.63 Å

RE-REFINED STRUCTURE OF THE HUMAN SIRT2 APOFORM

Assembly Summary

Multimeric state	2	Surface area, Å^2	27787.3	ΔG^{int}, kcal/mol	-14.5	$T\Delta S^{diss}$, kcal/mol	14.2
Copies in unit cell	8	Buried area, Å^2	6210.6	ΔG^{diss}, kcal/mol	23.5	Symmetry number	2
Formula	A_2ab_5c					Biomolecule (R350)	–
Composition	AC[P6G][PGE]$_5$[EDO]						
Dissociation pattern	A[P6G][PGE] + C[PGE]$_4$[EDO]						

View Dissociated | Download Assembly | Remark 350 | View | XML

Engaged interfaces

Id	##	Interfacing structures	N_{occ}	Diss.	Sym.ID	Buried area, Å^2	Δ^iG, kcal/mol	N_{HB}	N_{SB}	N_{DS}	CSS	
1	2	A + C	1	×	8_455	2339.5 (38%)	-31.9 (221%)	20 (87%)	7 (100%)	0	1.000	
						Average:	2414.1 (39%)	-31.5 (218%)	20 (87%)	10 (143%)	0	1.000
11	13	[PGE]C:1359 + C	1		1_555	142.7 (2%)	4.3 (30%)	0 (0%)	0 (0%)	0	0.000	
16	18	[PGE]C:1358 + C	1	×	1_555	121.4 (2%)	2.5 (17%)	0 (0%)	0 (0%)	0	0.000	
17	19	[PGE]C:1361 + C	1		4_555	116.2 (2%)	2.9 (20%)	0 (0%)	0 (0%)	0	0.000	
18	20	[EDO]A:1359 + A	1	×	1_555	100.5 (2%)	2.1 (14%)	1 (4%)	0 (0%)	0	0.000	
21	23	[PGE]A:1358 + C	1		1_455	90.1 (1%)	1.0 (7%)	0 (0%)	0 (0%)	0	0.000	
24	26	[PGE]C:1360 + C	1		1_555	77.1 (1%)	2.0 (14%)	1 (4%)	0 (0%)	0	0.000	
26	28	A + [PGE]C:1358	1		8_455	68.9 (1%)	1.3 (9%)	1 (4%)	0 (0%)	0	0.000	
30	32	[EDO]A:1359 + C	1		8_455	27.7 (0%)	0.1 (1%)	0 (0%)	0 (0%)	0	0.000	
31	33	[P6G]A:1357 + A	1		3_655	21.2 (0%)	1.3 (9%)	0 (0%)	0 (0%)	0	0.000	

View | Details | XML

Interface Summary XML

	Structure 1		Structure 2	
Selection range	A		C	
class	Protein		Protein	
symmetry operation	x,y,z		x-1/2,-y+1/2,-z	
symmetry ID	1_555		8_455	
Number of atoms				
interface	235	9.3%	251	10.4%
surface	1513	60.0%	1415	58.8%
total	2522	100.0%	2407	100.0%
Number of residues				
interface	66	20.8%	69	22.8%
surface	298	93.7%	283	93.4%
total	318	100.0%	303	100.0%
Solvent-accessible area, Å				
interface	2378.1	14.1%	2300.9	15.4%
total	16824.0	100.0%	14981.4	100.0%
Solvation energy, kcal/mol				
isolated structure	-309.0	100.0%	-308.3	100.0%
gain on complex formation	-15.0	4.8%	-16.9	5.5%
average gain	-7.0	2.3%	-5.7	1.8%
P-value	0.038		0.006	

View structure 1 | interface | structure 2

Download structure 1 | interface | structure 2

This interface scored

1.000

in Complex Formation Significance Score (CSS).

CSS ranges from 0 to 1 as interface relevance to complex formation increases.

Achieved CSS implies that the interface plays an essential role in complex formation

Hydrogen bonds XML

##	Structure 1	Dist. [Å]	Structure 2
1	A:ARG 42[NH2]	3.56	C:GLU 120[OE2]
2	A:ALA 85[N]	2.84	C:SER 293[O]
3	A:ARG 97[NH1]	2.90	C:GLN 265[OE1]
4	A:ARG 97[NH1]	2.96	C:ALA 290[O]
5	A:SER 100[OG]	2.24	C:ASP 308[OD2]
6	A:HIS 187[NE2]	2.67	C:ASP 294[OD2]
7	A:SER 263[N]	2.98	C:SER 293[OG]
8	A:SER 311[OG]	2.75	C:SER 100[OG]
9	A:LYS 312[N]	3.72	C:SER 100[OG]
10	A:LYS 313[N]	3.65	C:SER 100[OG]
11	A:LYS 313[NZ]	2.76	C:PRO 99[O]
12	A:LYS 313[NZ]	2.85	C:ASP 105[OD1]
13	A:GLU 108[OE2]	3.09	C:GLN 54[NE2]
14	A:GLN 265[OE1]	3.15	C:ARG 97[NH1]
15	A:ALA 290[O]	2.93	C:ARG 97[NH1]
16	A:GLU 35[OE1]	2.99	C:LYS 126[NZ]
17	A:ASP 294[OD1]	2.60	C:GLN 167[NE2]
18	A:ASP 294[OD1]	2.59	C:HIS 187[NE2]
19	A:ASP 95[OD1]	3.38	C:GLN 292[NE2]
20	A:ARG 97[O]	2.93	C:MET 307[N]

Salt bridges XML

##	Structure 1	Dist. [Å]	Structure 2
1	A:ARG 42[NH2]	3.56	C:GLU 120[OE2]
2	A:HIS 187[NE2]	2.67	C:ASP 294[OD2]
3	A:HIS 187[NE2]	3.22	C:ASP 294[OD1]
4	A:LYS 313[NZ]	2.85	C:ASP 105[OD1]
5	A:GLU 35[OE1]	2.99	C:LYS 126[NZ]
6	A:ASP 294[OD1]	2.59	C:HIS 187[NE2]
7	A:ASP 294[OD2]	3.25	C:HIS 187[NE2]

Figure S8. Synthetic peptide containing residues 286-313 of SIRT2 had no effect on the deacetylase or demyristoylase activities of $SIRT2^{cat}$. These assays were performed at 37°C and the concentration of NAD^+ was 1 mM. For the deacetylase assays (panel A), the $SIRT2^{cat}$ concentration was 40 nM and the acetyl peptide concentration was 4.4 μM. For the demyristoylase assays (panel B), the $SIRT2^{cat}$ concentration was 50 nM and the myristoyl peptide concentration was 500 nM.

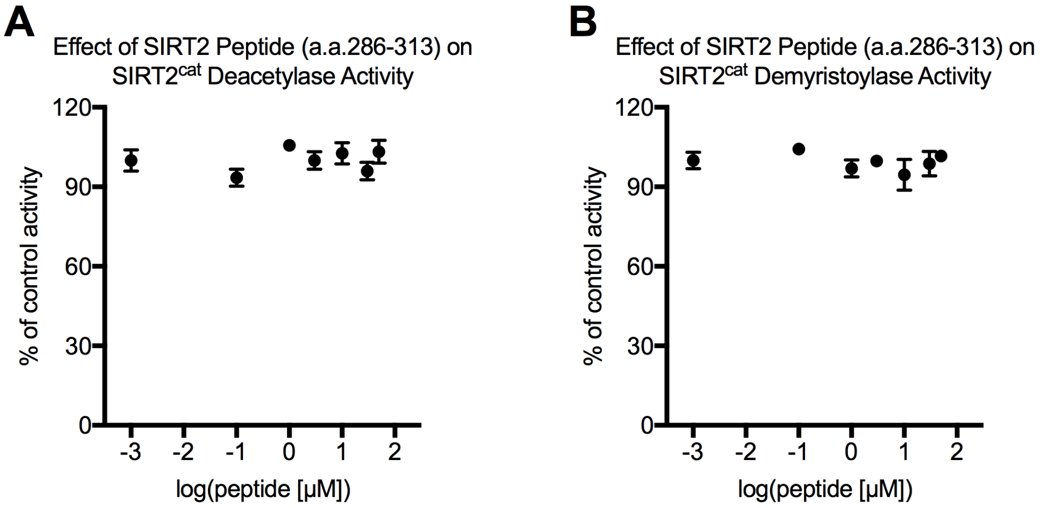

Figure S9. Detection of SIRT2 dimerization in cells, as in Figure 4 of the main article, but with different time points post-transfection. (A) Fluorescence imaging of A549 cells expressing the split GFP fragment-tagged SIRT2 proteins 24 hours after transfection. (B) Quantification of the GFP fluorescence intensities from the images shown in panel A. The GFP fluorescence intensities from individual cells were normalized to the DAPI fluorescence intensities from the same cells. Panels (C) and (D) are the same as (A) and (B), except a 72 hour time point post-transfection was used. The relative fluorescence intensities were compared with a Student's *t*-test (**, $p < 0.01$; ***, $p < 0.001$).

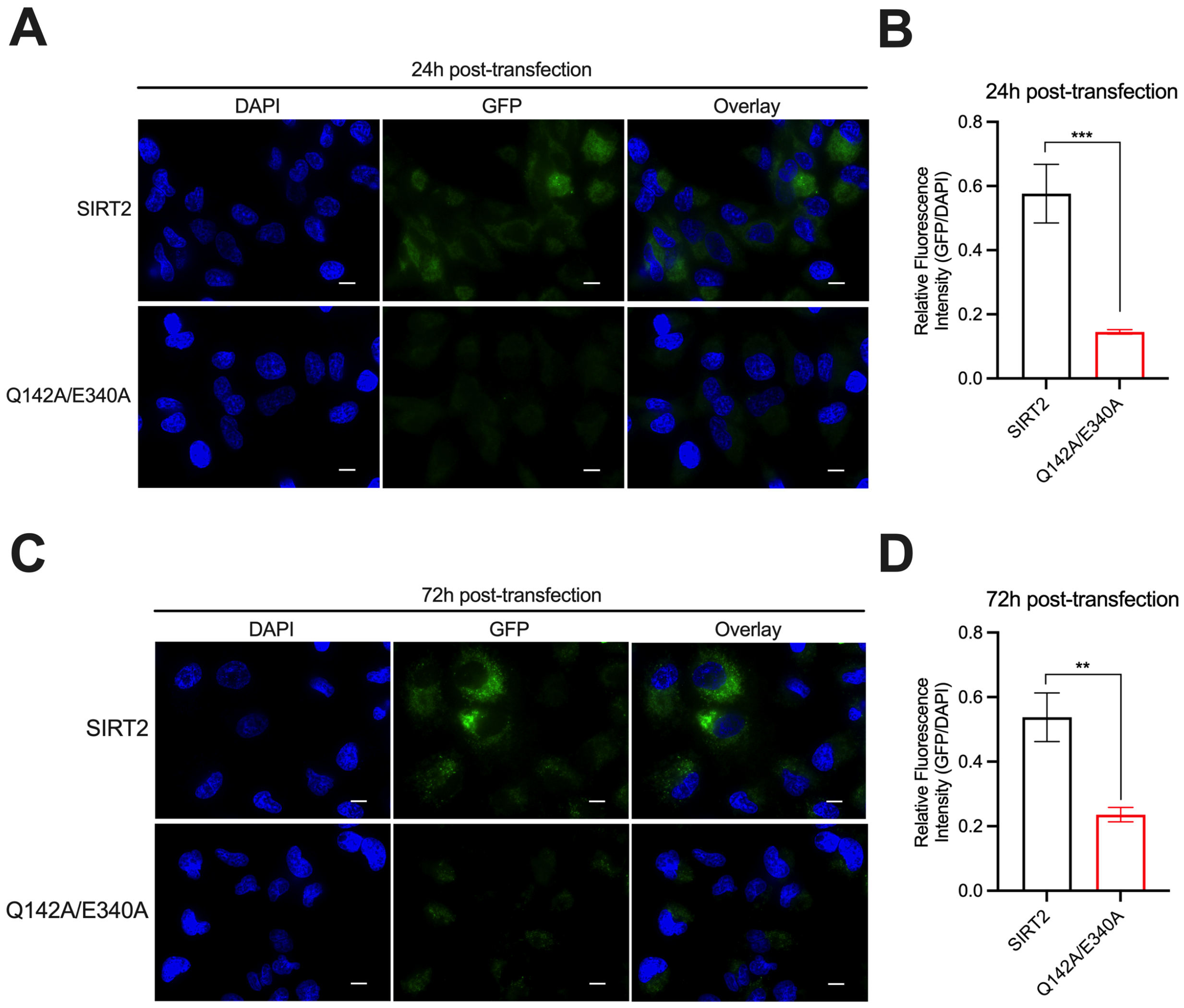

Supporting Information Methods

Prediction of SIRT2 Oligomeric States Based on X-ray Crystal Structures – Table S1

To obtain the PDB codes, a search query was performed in the Protein Databank[23] (January 2023) using 135 internal residues from the sequence of the SIRT2 catalytic domain. The PDB codes were individually put into the PDBePISA webserver which reported the most probable or predicted biological assembly.[1] When the most probable assembly was a multimer, the structure was downloaded and compared to the structure in its .pdb file, also obtained from Protein Databank, depicting the asymmetric unit.

SIRT2 Binding Assay with Cy3-myristoyl-H4K16-peptide – Figure S3

Assays were performed at 23°C in buffer containing PBS and 1 mM DTT. A quartz microcuvette was used along with the Horiba Fluoromax-4 instrument to measure fluorescence intensity. The total volume for each measurement was 160 µl, and the excitation/emission wavelengths were 535/560 nm.

We measured the interaction of SIRT2 with Cy3-myristoyl-H4K16 peptide using fluorescence intensity measurements essentially as described.[22,24] Briefly, background fluorescence was first measured for the given concentration of SIRT2 protein in a cuvette, then 1 µl of the fluorescent peptide was added to a final concentration of 50 nM. After mixing, the fluorescence was again measured, and the background fluorescence was subtracted from the values in the presence of fluorescent peptide. The background-subtracted fluorescence values were used in the binding curves, and where applicable, competitor unlabeled peptides were included in the cuvette during background measurements. The equation to fit a quadratic binding curve (to quantify SIRT2 interaction with Cy3-myristoyl-H4K16 peptide) and the equation to fit the sigmoidal curve to the competition data was reported in our previous work;[24,25] for the data shown in this article, a standard Hill slope of 1 was used for the sigmoidal curve.

SIRT2/Peptide EMSA – Figure S5

Native PAGE EMSA experiments were run on Bio-Rad 4-15% polyacrylamide Tris-glycine gels with a running buffer of 25 mM Tris and 192 mM glycine. Protein was separated on the gels for 1 hour at 110 V with the protein migrating towards the positive electrode. The protein and protein/peptide mixtures were in PBS with 8% glycerol prior to loading on the gels without any additional loading buffer. The protein/peptide mixtures were equilibrated for ~5 minutes at room temperature prior to loading on the gel. After electrophoresis, the native gels were scanned with an Azure c400 fluorescent imager using excitation/emission filters designed for green fluorescence. The gel was then stained with Coomassie blue and imaged under white light. Gel band intensity from the Coomassie gels was quantified with Fiji/ImageJ to determine the fraction of SIRT2 bound to peptide at each peptide concentration. This data was plotted, and a curve was fit using the standard quadratic equation to determine K_d values for protein-peptide binding.[24,25]

X-ray crystallography Methods

SIRT2 catalytic domain (amino acids 34-356) was purified essentially as described.[24] For our final gel filtration column, we used a mobile phase of 20 mM Tris-Cl (pH 7.4), 75 mM NaCl, and 0.1 mM DTT. After eluting off the column, the protein was immediately concentrated to ~11.5 mg/ml, then FAM-myristoyl-H4K16 peptide dissolved at 3.9 mg/ml in an identical buffer

was added such that the final molar ratio of protein to peptide was 1 to 4. Material was aliquoted then snap frozen in liquid N_2. Upon thawing, the protein was tested in a series of commercially available sparse matrix crystallization screens using a Douglas Instruments Oryx drop-setter robot. The drops were 0.2 µl protein mixed with 0.2 µl reservoir solution, and the reservoir volumes were 30 µl. The protein/peptide complex formed crystals over a reservoir of 0.2 M MES monohydrate pH 6.5 (NaOH), 20% w/v PEG 4,000.

Crystals were cryo-protected with 20% ethanediol in reservoir solution then flash frozen in liquid nitrogen. Diffraction data was collected at the SER-CAT (APS sector 22) ID hutch using an incident beam of 1.0 Å wavelength and an Eiger 16M pixel array detector. Use of the Advanced Photon Source was supported by the U. S. Department of Energy, Office of Science, Office of Basic Energy Sciences, under Contract No. W-31-109-Eng-38. Datasets were reduced using HKL-2000 in the space group P2.[26]

The structure was phased in Phaser as part of the Phenix software suite.[27,28] The search model was drawn from PDB code 5FYQ.[12] Calculation of the Matthews coefficient indicated one protein monomer in the asymmetric unit with a solvent content of 45.70%.[29,30] A solution was found in space group $P2_1$. The model was put through rounds of manual rebuilding in Coot and reciprocal space refinement in Phenix.[31,32] The parameter file for the synthetic peptide's myristoyl group was built in Elbow.[33] Representations of the crystal structure were created in PyMOL.[34]

Sequences of split GFP fragment-fused SIRT2 proteins used in Figures 4 and 5 of the main article

Color code: GFP fragment ; linker region ; SIRT2 ; point mutation

AA sequence for GFP-Nterm-SIRT2

MGHHHHHHGGASKGERLFRGKVPILVELKGDVNGHKFSVRGEGKGDATRGKLTLKFIC
TTGKLPVPWPTLVTTLTYGVQCFSRYPKHMKRHDFFKSAMPKGYVQERTISFKKDGKY
KTRAEVKFEGRTLVNRIKLKGRDFKEKGNILGHKLRYNFNSHKVYITADKRGGSGSGSS
MAEPDPSHPLETQAGKVQEAQDSDSDSEGGAAGGEADMDFLRNLFSQTLSLGSQKERL
LDELTLEGVARYMQSERCRRVICLVGAGISTSAGIPDFRSPSTGLYDNLEKYHLPYPEAIF
EISYFKKHPEPFFALAKELYPGQFKPTICHYFMRLLKDKGLLLRCYTQNIDTLERIAGLEQ
EDLVEAHGTFYTSHCVSASCRHEYPLSWMKEKIFSEVTPKCEDCQSLVKPDIVFFGESLP
ARFFSCMQSDFLKVDLLLVMGTSLQVQPFASLISKAPLSTPRLLINKEKAGQSDPFLGMI
MGLGGGMDFDSKKAYRDVAWLGECDQGCLALAELLGWKKELEDLVRREHASIDAQS
GAGVPNPSTSASPKKSPPPAKDEARTTEREKPQ

AA sequence for SIRT2-GFP-Cterm

MAEPDPSHPLETQAGKVQEAQDSDSDSEGGAAGGEADMDFLRNLFSQTLSLGSQKERL
LDELTLEGVARYMQSERCRRVICLVGAGISTSAGIPDFRSPSTGLYDNLEKYHLPYPEAIF
EISYFKKHPEPFFALAKELYPGQFKPTICHYFMRLLKDKGLLLRCYTQNIDTLERIAGLEQ
EDLVEAHGTFYTSHCVSASCRHEYPLSWMKEKIFSEVTPKCEDCQSLVKPDIVFFGESLP
ARFFSCMQSDFLKVDLLLVMGTSLQVQPFASLISKAPLSTPRLLINKEKAGQSDPFLGMI
MGLGGGMDFDSKKAYRDVAWLGECDQGCLALAELLGWKKELEDLVRREHASIDAQS

GAGVPNPSTSASPKKSPPPAKDEARTTEREKPQTSGGSGKNGIKAKFKIRHNVKDGSVQ
LADHYQQNTPIGRGPVLLPRNHYLSTRSKLSKDPKEKRDHMVLLEFVTAAGIKHGRDER
YK

AA sequence for GFP-Nterm-SIRT2(Q142A/E340A)
MGHHHHHHGGASKGERLFRGKVPILVELKGDVNGHKFSVRGEGKGDATRGKLTLKFIC
TTGKLPVPWPTLVTTLTYGVQCFSRYPKHMKRHDFFKSAMPKGYVQERTISFKKDGKY
KTRAEVKFEGRTLVNRIKLKGRDFKEKGNILGHKLRYNFNSHKVYITADKRGGSGSGSS
MAEPDPSHPLETQAGKVQEAQDSDSDSEGGAAGGEADMDFLRNLFSQTLSLGSQKERL
LDELTLEGVARYMQSERCRRVICLVGAGISTSAGIPDFRSPSTGLYDNLEKYHLPYPEAIF
EISYFKKHPEPFFALAKELYPGAFKPTICHYFMRLLKDKGLLLRCYTQNIDTLERIAGLEQ
EDLVEAHGTFYTSHCVSASCRHEYPLSWMKEKIFSEVTPKCEDCQSLVKPDIVFFGESLP
ARFFSCMQSDFLKVDLLLVMGTSLQVQPFASLISKAPLSTPRLLINKEKAGQSDPFLGMI
MGLGGGMDFDSKKAYRDVAWLGECDQGCLALAELLGWKKALEDLVRREHASIDAQS
GAGVPNPSTSASPKKSPPPAKDEARTTEREKPQ

AA sequence for SIRT2(Q142A/E340A)-GFP-Cterm
MAEPDPSHPLETQAGKVQEAQDSDSDSEGGAAGGEADMDFLRNLFSQTLSLGSQKERL
LDELTLEGVARYMQSERCRRVICLVGAGISTSAGIPDFRSPSTGLYDNLEKYHLPYPEAIF
EISYFKKHPEPFFALAKELYPGAFKPTICHYFMRLLKDKGLLLRCYTQNIDTLERIAGLEQ
EDLVEAHGTFYTSHCVSASCRHEYPLSWMKEKIFSEVTPKCEDCQSLVKPDIVFFGESLP
ARFFSCMQSDFLKVDLLLVMGTSLQVQPFASLISKAPLSTPRLLINKEKAGQSDPFLGMI
MGLGGGMDFDSKKAYRDVAWLGECDQGCLALAELLGWKKALEDLVRREHASIDAQS
GAGVPNPSTSASPKKSPPPAKDEARTTEREKPQTSGGSGKNGIKAKFKIRHNVKDGSVQ
LADHYQQNTPIGRGPVLLPRNHYLSTRSKLSKDPKEKRDHMVLLEFVTAAGIKHGRDER
YK

AA sequence for GFP-Nterm fragment (not fused to SIRT2)
MGHHHHHHGGASKGERLFRGKVPILVELKGDVNGHKFSVRGEGKGDATRGKLTLKFIC
TTGKLPVPWPTLVTTLTYGVQCFSRYPKHMKRHDFFKSAMPKGYVQERTISFKKDGKY
KTRAEVKFEGRTLVNRIKLKGRDFKEKGNILGHKLRYNFNSHKVYITADKRGGSGSGSS

AA sequence for GFP-Cterm fragment (not fused to SIRT2)
MTSGGSGKNGIKAKFKIRHNVKDGSVQLADHYQQNTPIGRGPVLLPRNHYLSTRSKLSK
DPKEKRDHMVLLEFVTAAGIKHGRDERYK

Supporting Information References

(1) Krissinel, E., and Henrick, K. (2007) Inference of macromolecular assemblies from crystalline state. *J Mol Biol 372*, 774–797.
(2) Finnin, M. S., Donigian, J. R., and Pavletich, N. P. (2001) Structure of the histone deacetylase SIRT2. *Nat. Struct. Biol. 8*, 621–625.
(3) Moniot, S., Schutkowski, M., and Steegborn, C. (2013) Crystal structure analysis of human Sirt2 and its ADP-ribose complex. *J. Struct. Biol. 182*, 136–143.
(4) Yamagata, K., Goto, Y., Nishimasu, H., Morimoto, J., Ishitani, R., Dohmae, N., Takeda, N., Nagai, R., Komuro, I., Suga, H., and Nureki, O. (2014) Structural Basis for Potent Inhibition of SIRT2 Deacetylase by a Macrocyclic Peptide Inducing Dynamic Structural Change. *Structure 22*, 345–352.
(5) Rumpf, T., Schiedel, M., Karaman, B., Roessler, C., North, B. J., Lehotzky, A., Oláh, J., Ladwein, K. I., Schmidtkunz, K., Gajer, M., Pannek, M., Steegborn, C., Sinclair, D. A., Gerhardt, S., Ovádi, J., Schutkowski, M., Sippl, W., Einsle, O., and Jung, M. (2015) Selective Sirt2 inhibition by ligand-induced rearrangement of the active site. *Nat Commun 6*, 6263.
(6) Teng, Y.-B., Jing, H., Aramsangtienchai, P., He, B., Khan, S., Hu, J., Lin, H., and Hao, Q. (2015) Efficient demyristoylase activity of SIRT2 revealed by kinetic and structural studies. *Sci Rep 5*, 8529.
(7) Rumpf, T., Gerhardt, S., Einsle, O., and Jung, M. (2015) Seeding for sirtuins: microseed matrix seeding to obtain crystals of human Sirt3 and Sirt2 suitable for soaking. *Acta Crystallogr F Struct Biol Commun 71*, 1498–1510.
(8) Feldman, J. L., Dittenhafer-Reed, K. E., Kudo, N., Thelen, J. N., Ito, A., Yoshida, M., and Denu, J. M. (2015) Kinetic and Structural Basis for Acyl-Group Selectivity and NAD(+) Dependence in Sirtuin-Catalyzed Deacylation. *Biochemistry 54*, 3037–3050.
(9) Wang, Y., Fung, Y. M. E., Zhang, W., He, B., Chung, M. W. H., Jin, J., Hu, J., Lin, H., and Hao, Q. (2017) Deacylation Mechanism by SIRT2 Revealed in the 1'-SH-2'-O-Myristoyl Intermediate Structure. *Cell Chem Biol 24*, 339–345.
(10) Schiedel, M., Rumpf, T., Karaman, B., Lehotzky, A., Oláh, J., Gerhardt, S., Ovádi, J., Sippl, W., Einsle, O., and Jung, M. (2016) Aminothiazoles as Potent and Selective Sirt2 Inhibitors: A Structure-Activity Relationship Study. *J Med Chem 59*, 1599–1612.
(11) Schiedel, M., Rumpf, T., Karaman, B., Lehotzky, A., Gerhardt, S., Ovádi, J., Sippl, W., Einsle, O., and Jung, M. (2016) Structure-Based Development of an Affinity Probe for Sirtuin 2. *Angew Chem Int Ed Engl 55*, 2252–2256.
(12) Knyphausen, P., de Boor, S., Kuhlmann, N., Scislowski, L., Extra, A., Baldus, L., Schacherl, M., Baumann, U., Neundorf, I., and Lammers, M. (2016) Insights into Lysine Deacetylation of Natively Folded Substrate Proteins by Sirtuins. *J. Biol. Chem. 291*, 14677–14694.
(13) Sundriyal, S., Moniot, S., Mahmud, Z., Yao, S., Di Fruscia, P., Reynolds, C. R., Dexter, D. T., Sternberg, M. J. E., Lam, E. W.-F., Steegborn, C., and Fuchter, M. J. (2017) Thienopyrimidinone Based Sirtuin-2 (SIRT2)-Selective Inhibitors Bind in the Ligand Induced Selectivity Pocket. *J Med Chem 60*, 1928–1945.
(14) Moniot, S., Forgione, M., Lucidi, A., Hailu, G. S., Nebbioso, A., Carafa, V., Baratta, F., Altucci, L., Giacché, N., Passeri, D., Pellicciari, R., Mai, A., Steegborn, C., and Rotili, D. (2017) Development of 1,2,4-Oxadiazoles as Potent and Selective Inhibitors of the Human Deacetylase

Sirtuin 2: Structure-Activity Relationship, X-ray Crystal Structure, and Anticancer Activity. *J. Med. Chem. 60*, 2344–2360.
(15) Jin, J., He, B., Zhang, X., Lin, H., and Wang, Y. (2016) SIRT2 Reverses 4-Oxononanoyl Lysine Modification on Histones. *J. Am. Chem. Soc. 138*, 12304–12307.
(16) Mellini, P., Itoh, Y., Tsumoto, H., Li, Y., Suzuki, M., Tokuda, N., Kakizawa, T., Miura, Y., Takeuchi, J., Lahtela-Kakkonen, M., and Suzuki, T. (2017) Potent mechanism-based sirtuin-2-selective inhibition by an in situ-generated occupant of the substrate-binding site, "selectivity pocket" and NAD+-binding site. *Chem Sci 8*, 6400–6408.
(17) Kudo, N., Ito, A., Arata, M., Nakata, A., and Yoshida, M. (2018) Identification of a novel small molecule that inhibits deacetylase but not defatty-acylase reaction catalysed by SIRT2. *Philos Trans R Soc Lond B Biol Sci 373*, 20170070.
(18) Yang, L.-L., Wang, H.-L., Zhong, L., Yuan, C., Liu, S.-Y., Yu, Z.-J., Liu, S., Yan, Y.-H., Wu, C., Wang, Y., Wang, Z., Yu, Y., Chen, Q., and Li, G.-B. (2018) X-ray crystal structure guided discovery of new selective, substrate-mimicking sirtuin 2 inhibitors that exhibit activities against non-small cell lung cancer cells. *European Journal of Medicinal Chemistry 155*, 806–823.
(19) You, W., Zheng, W., Weiss, S., Chua, K. F., and Steegborn, C. (2019) Structural basis for the activation and inhibition of Sirtuin 6 by quercetin and its derivatives. *Sci Rep 9*, 19176.
(20) Hong, J. Y., Price, I. R., Bai, J. J., and Lin, H. (2019) A Glycoconjugated SIRT2 Inhibitor with Aqueous Solubility Allows Structure-Based Design of SIRT2 Inhibitors. *ACS Chem Biol 14*, 1802–1810.
(21) Nielsen, A. L., Rajabi, N., Kudo, N., Lundø, K., Moreno-Yruela, C., Bæk, M., Fontenas, M., Lucidi, A., Madsen, A. S., Yoshida, M., and Olsen, C. A. (2021) Mechanism-based inhibitors of SIRT2: structure-activity relationship, X-ray structures, target engagement, regulation of α-tubulin acetylation and inhibition of breast cancer cell migration. *RSC Chem Biol 2*, 612–626.
(22) Hong, J. Y., Cassel, J., Yang, J., Lin, H., and Weiser, B. P. (2021) High-Throughput Screening Identifies Ascorbyl Palmitate as a SIRT2 Deacetylase and Defatty-Acylase Inhibitor. *ChemMedChem 16*, 3484–3494.
(23) Berman, H. M., Westbrook, J., Feng, Z., Gilliland, G., Bhat, T. N., Weissig, H., Shindyalov, I. N., and Bourne, P. E. (2000) The Protein Data Bank. *Nucleic Acids Res 28*, 235–242.
(24) Bi, D., Yang, J., Hong, J. Y., Parikh, P., Hinds, N., Infanti, J., Lin, H., and Weiser, B. P. (2020) Substrate-Dependent Modulation of SIRT2 by a Fluorescent Probe, 1-Aminoanthracene. *Biochemistry 59*, 3869–3878.
(25) Weiser, B. P., Stivers, J. T., and Cole, P. A. (2017) Investigation of N-Terminal Phospho-Regulation of Uracil DNA Glycosylase Using Protein Semisynthesis. *Biophys. J. 113*, 393–401.
(26) Otwinowski, Z., and Minor, W. (1997) [20] Processing of X-ray diffraction data collected in oscillation mode, in *Methods in Enzymology*, pp 307–326. Academic Press.
(27) McCoy, A. J., Grosse-Kunstleve, R. W., Adams, P. D., Winn, M. D., Storoni, L. C., and Read, R. J. (2007) Phaser crystallographic software. *J Appl Crystallogr 40*, 658–674.
(28) Liebschner, D., Afonine, P. V., Baker, M. L., Bunkóczi, G., Chen, V. B., Croll, T. I., Hintze, B., Hung, L. W., Jain, S., McCoy, A. J., Moriarty, N. W., Oeffner, R. D., Poon, B. K., Prisant, M. G., Read, R. J., Richardson, J. S., Richardson, D. C., Sammito, M. D., Sobolev, O. V., Stockwell, D. H., Terwilliger, T. C., Urzhumtsev, A. G., Videau, L. L., Williams, C. J., and Adams, P. D. (2019) Macromolecular structure determination using X-rays, neutrons and electrons: recent developments in Phenix. *Acta Crystallogr D Struct Biol 75*, 861–877.

(29) Matthews, B. W. (1968) Solvent content of protein crystals. *Journal of Molecular Biology 33*, 491–497.
(30) Weichenberger, C. X., and Rupp, B. (2014) Ten years of probabilistic estimates of biocrystal solvent content: new insights via nonparametric kernel density estimate. *Acta Crystallogr D Biol Crystallogr 70*, 1579–1588.
(31) Emsley, P., Lohkamp, B., Scott, W. G., and Cowtan, K. (2010) Features and development of Coot. *Acta Crystallogr D Biol Crystallogr 66*, 486–501.
(32) Afonine, P. V., Grosse-Kunstleve, R. W., Echols, N., Headd, J. J., Moriarty, N. W., Mustyakimov, M., Terwilliger, T. C., Urzhumtsev, A., Zwart, P. H., and Adams, P. D. (2012) Towards automated crystallographic structure refinement with phenix.refine. *Acta Crystallogr D Biol Crystallogr 68*, 352–367.
(33) Moriarty, N. W., Grosse-Kunstleve, R. W., and Adams, P. D. (2009) electronic Ligand Builder and Optimization Workbench (eLBOW): a tool for ligand coordinate and restraint generation. *Acta Crystallogr D Biol Crystallogr 65*, 1074–1080.
(34) The PyMOL Molecular Graphics System, Version 1.5.0.4 Schrödinger, LLC.
